# Supplementary material for: National administrative record linkage between specialist community drug and alcohol treatment data (the National Drug Treatment Monitoring System (NDTMS)) and inpatient hospitalisation data (Hospital Episode Statistics (HES)) in England: design, method and evaluation
Source: BMJ Open. 2020 Nov 26;10(11):e043540. doi: 10.1136/bmjopen-2020-043540 (PMC7692978; doi:10.1136/bmjopen-2020-043540)
Supplement: Supplementary data [file bmjopen-2020-043540supp001.pdf]

Online Supplementary Material

Contents

Tables

S1: Differences between full NDTMS cohort and the 'gold-standard' NDTMS cohort with NHS number available

S2: The odds of an average ≥ 5 day length of hospital admission within the 'gold-standard' NDTMS cohort with NHS number available, in the 'unbiased' sample matched using NHS number and the 'biased' sample matched using the four-stage algorithm, following application of inverse probability weighting to correct for potential linkage bias

S3: Differences between NDTMS cohort that contains sufficient matching data (i.e. a validly coded dob, sex and postcode) and the NDTMS cohort that does not contain sufficient matching data

S4: Description of variables available for linkage

References

Tables

Table S1: Differences between full NDTMS cohort and the 'gold-standard' NDTMS cohort with NHS number available

		Full NDTMS Cohort n (%)	Cohort with NHS number n (%)	p value
**All**		268,251 (100.0)	1,328 (100.0)	
**Sociodemographic**	**Sex**			
	Female	83,015 (31.0)	393 (29.6)	0.29
	Male	185,236 (69.0)	935 (70.4)	
	**Age in years (at presentation to D&A services)**			
	18-30	36,068 (13.4)	143 (10.8)	0.04*
	31-45	127,635 (47.6)	720 (54.2)	
	46-60	89,643 (33.4)	438 (33.0)	
	60+	14,905 (5.6)	27 (2.0)	
	**Deprivation (IMD) Quintile**			
	First (Most deprived)	81,478 (33.2)	472 (39.9)	<0.001*
	Second	68,808 (28.0)	366 (29.4)	
	Third	47,139 (19.2)	271 (21.8)	
	Fourth	33,354 (13.6)	111 (8.9)	
	Fifth (Least deprived)	15,002 (6.1)	25 (2.0)	
	**Residential Status**			
	Non NFA postcode	255,515 (95.3)	1,286 (96.8)	0.01*
	NFA postcode	12,736 (4.7)	42 (3.2)	
	**Ethnicity**[1]			
	White	230,012 (90.6)	1,064 (87.5)	<0.001*
	Non-white	23,928 (9.4)	152 (12.5)	
**Clinical**	**Substance Misuse**[2]			
	Opioid	139,845 (52.1)	775 (58.4)	<0.001*
	Alcohol only	75,555 (28.2)	362 (27.3)	
	Non-opioid and alcohol	28,598 (10.7)	95 (7.1)	
	Non-opioid only	24,253 (9.0)	96 (7.2)	

* p<0.05; 1 Office of Population Censuses and Surveys (OPCS) categories A, B and C collapsed as white, all other OPCS categories (D-S) collapsed as non-white
2 NDTMS categorisation **opioid**: clients with any mention of opioid use in any treatment episode during the year irrespective of other substances cited; **alcohol only:** clients who present with problems related to alcohol but no other substances; **non-opioid and alcohol:** clients with non-opioid drug *and* alcohol use problems (but *not* opioids) recorded in any treatment episode during the year; **non-opioid only:** clients who present for treatment related to non-opioid drug use but *not* opioids or alcohol

Table S2: The conditional odds of an average ≥ 5 day length of hospital admission within the 'gold-standard' NDTMS cohort with NHS number available, in the 'unbiased' sample matched using NHS number and the 'biased' sample matched using the four-stage algorithm, following application of inverse probability weighting to correct for potential linkage bias

		'Unbiased' sample matched using NHS number		'Biased' sample matched using four-stage algorithm		
		n (%)	OR (95%CI)	n (%)	OR (95%CI)	Weighted OR[1] (95%CI)
	**All**	1,153 (100.0)	-	1,053 (100.0)	-	-
**Sociodemographic**	**Sex**					
	Female	370 (32.1)	Reference	336 (31.9)	Reference	Reference
	Male	783 (67.9)	1.27 (0.80-2.02)	717 (68.1)	1.33 (0.85-2.09)	1.26 (0.80-2.00)
	**Age in years (at presentation to D&A services)**					
	≤ 45	747 (64.8)	Reference	658 (62.5)	Reference	Reference
	> 45	406 (35.2)	1.07 (0.69-1.65)	395 (37.5)	1.17 (0.78-1.77)	1.11 (0.71-1.73)
	**Deprivation (IMD) Quintile**					
	First (Most deprived)	402 (37.4)	Reference	363 (35.7)	Reference	Reference
	All other quintiles	674 (62.6)	0.64 (0.42-1.00)	655 (64.3)	0.71 (0.47-1.08)	0.70 (0.40-1.10)
	**Residential Status**					
	Non NFA postcode	1,115 (96.7)	Reference	1,011 (96.0)	Reference	Reference
	NFA postcode	38 (3.3)	0.59 (0.13-2.51)	42 (4.0)	0.56 (0.13-2.35)	0.60 (0.12-2.49)
	**Ethnicity[2]**					
	White	945 (89.7)	Reference	915 (90.0)	Reference	Reference
	Non-white	109 (10.3)	1.74 (0.90-3.36)	102 (10.0)	2.19 (1.25-3.84)	1.89 (0.99-3.39)
**Clinical**	**Substance Misuse[3]**					
	Opioid	651 (56.5)	Reference	569 (54.0)	Reference	Reference
	All other drug categories	502 (43.5)	0.67 (0.43-1.04)	484 (46.0)	0.65 (0.43-0.99)	0.65 (0.42-1.00)

OR Odds Ratio; CI Confidence Interval; IMD Index of Multiple Deprivation

1 Model with inverse probability weighting for matching included; 2 Office of Population Censuses and Surveys (OPCS) categories A, B and C collapsed as white, all other OPCS categories (D-S) collapsed as non-white; 3 NDTMS categorisation **opioid**: clients with any mention of opioid use in any treatment episode during the year irrespective of other substances cited; **alcohol only:** clients who present with problems related to alcohol but no other substances; **non-opioid and alcohol:** clients with non-opioid drug *and* alcohol use problems (but *not* opioids) recorded in any treatment episode during the year; **non-opioid only:** clients who present for treatment related to non-opioid drug use but *not* opioids or alcohol

Table S3: Differences between NDTMS cohort that contains sufficient matching data (i.e. a validly coded dob, sex and postcode) and the NDTMS cohort that does not contain sufficient matching data

		NDTMS cohort that does contain sufficient matching data n (%)	NDTMS cohort that does not contain sufficient matching data n (%)	OR (95%CI)	aOR (95%CI)[1]
**All**		258,240 (100.0)	10,011 (100.0)	-	
**Sociodemographic**	**Sex**				
	Female	80,111 (31.0)	2,904 (29.0)	Reference	Reference
	Male	178,129 (69.0)	7,107 (71.0)	1.10 (1.05-1.15)	1.27 (1.15-1.40)
	**Age in years (at presentation to D&A services)**				
	18-30	35,446 (13.7)	622 (6.2)	Reference	Reference
	31-45	122,999 (47.6)	4,636 (56.3)	2.15 (1.97-2.34)	0.94 (0.83-1.06)
	46-60	85,425 (33.1)	4,218 (42.1)	2.81 (2.58-3.06)	0.61 (0.53-0.71)
	60+	14,370 (5.6)	535 (5.3)	2.12 (1.89-2.39)	0.52 (0.40-0.67)
	**Deprivation (IMD) Quintile[2]**				
	First (Most deprived)	81,478 (33.2)	-	-	-
	Second	68,808 (28.0)	-	-	-
	Third	47,139 (19.2)	-	-	-
	Fourth	33,354 (13.6)	-	-	-
	Fifth (Least deprived)	15,002 (6.1)	-	-	-
	**Residential Status[2]**				-
	Non NFA postcode	245,504 (95.1)	-	-	-
	NFA postcode	12,736 (4.9)	-	-	-
	**Ethnicity[3]**				
	White	228,023 (90.6)	1,989 (89.2)	Reference	Reference
	Non-white	23,688 (9.4)	240 (10.8)	1.16 (1.02-1.33)	0.84 (0.73-0.97)
**Clinical**	**Substance Misuse[4]**				
	Opioid	131,316 (50.9)	8,529 (85.2)	Reference	Reference
	Alcohol only	74,840 (29.0)	715 (7.1)	0.15 (0.14-0.16)	0.86 (0.77-0.95)
	Non-opioid and alcohol	28,202 (10.9)	396 (4.0)	0.22 (0.20-0.24)	1.07 (0.93-1.22)
	Non-opioid only	23,882 (9.3)	371 (3.7)	0.24 (0.22-0.27)	1.05 (0.91-1.22)

1 Adjusted for all other covariates listed in table 2 All records that did not contain sufficient matching data lacked validly coded postcodes, as such no IMD quintile or residential status values were available for this cohort. 3 Office of Population Censuses and Surveys (OPCS) categories A, B and C collapsed as white, all other OPCS categories (D-S) collapsed as non-white 4 NDTMS categorisation **opioid**: clients with any mention of opioid use in any treatment episode during the year irrespective of other substances cited; **alcohol only:** clients who present with problems related to alcohol but no other substances; **non-opioid and alcohol:** clients with non-opioid drug *and* alcohol use problems (but *not* opioids) recorded in any treatment episode during the year; **non-opioid only:** clients who present for treatment related to non-opioid drug use but *not* opioids or alcohol

Table S4: Description of variables available for linkage

Variable	Format	Notes
Date of Birth (*dob*)	'yyyymmdd'	
Sex (*sex*)	1=male; 2=female	
Sector Level Postcode (*postcode*)	e.g. 'S752' or 'NE177'	This contains the entire 'outcode' and the first character of the 'incode', each unique sector level postcode representing roughly 3000 households in England (1)
Ethnicity (*ethnicity*)	e.g. 'A'	16 categories A - S harmonised to current Office of Population Censuses and Surveys (OPCS) definitions (2, 3)
GP Practice Code (*gppractice*)	e.g. 'C87034'	A unique six character code ascribed to each GP practice in England (3, 4)

References

1. https://www.mrs.org.uk/pdf/postcodeformat.pdf.
2. Harmonised country specific ethnic group question(s) and dissemination of output(s) for use in social surveys and administrative data in England, Northern Ireland, Scotland and Wales https://gss.civilservice.gov.uk/policy-store/ethnicity/#great-britain.
3. Hospital Episode Statistics Data Dictionary https://digital.nhs.uk/data-and-information/data-tools-and-services/data-services/hospital-episode-statistics/hospital-episode-statistics-data-dictionary.
4. GP and GP practice related data https://digital.nhs.uk/services/organisation-data-service/data-downloads/gp-and-gp-practice-related-data.
