# Supplementary material for: National administrative record linkage between specialist community drug and alcohol treatment data (the National Drug Treatment Monitoring System (NDTMS)) and inpatient hospitalisation data (Hospital Episode Statistics (HES)) in England: design, method and evaluation
Source: BMJ Open. 2020 Nov 26;10(11):e043540. doi: 10.1136/bmjopen-2020-043540 (PMC7692978; doi:10.1136/bmjopen-2020-043540)
Supplement: Author's manuscript [file bmjopen-2020-043540.draft_revisions.pdf]

BMJ Open

BMJ Open is committed to open peer review. As part of this commitment we make the peer review history of every article we publish publicly available.

When an article is published we post the peer reviewers' comments and the authors' responses online. We also post the versions of the paper that were used during peer review. These are the versions that the peer review comments apply to.

The versions of the paper that follow are the versions that were submitted during the peer review process. They are not the versions of record or the final published versions. They should not be cited or distributed as the published version of this manuscript.

BMJ Open is an open access journal and the full, final, typeset and author-corrected version of record of the manuscript is available on our site with no access controls, subscription charges or pay-per-view fees (http://bmjopen.bmj.com).

If you have any questions on BMJ Open's open peer review process please email info.bmjopen@bmj.com

BMJ Open

A national administrative record linkage between specialist community drug and alcohol treatment data (The National Drug Treatment Monitoring System (NDTMS)) and inpatient hospitalisation data (Hospital Episode Statistics (HES)) in England; Design, Method and Evaluation

Journal:	*BMJ Open*
Manuscript ID	bmjopen-2020-043540
Article Type:	Original research
Date Submitted by the Author:	06-Aug-2020

Complete List of Authors:	Roberts , Emmert; Institute of Psychiatry Psychology and Neuroscience, National Addiction Centre; South London and Maudsley NHS Foundation Trust,<br>Doidge, James<br>Harron, Katie; London School of Hygiene and Tropical Medicine,<br>Hotopf, Matthew; King's College London (Institute of Psychiatry),<br>Knight, Jonathan; Public Health England<br>White, Martin; Public Health England<br>Eastwood, Brian; Public Health England<br>Drummond, Colin; Kings College London, Institute of Psychiatry

Keywords:	PUBLIC HEALTH, EPIDEMIOLOGY, Substance misuse < PSYCHIATRY

SCHOLARONE™
Manuscripts

I, the Submitting Author has the right to grant and does grant on behalf of all authors of the Work (as defined in the below author licence), an exclusive licence and/or a non-exclusive licence for contributions from authors who are: i) UK Crown employees; ii) where BMJ has agreed a CC-BY licence shall apply, and/or iii) in accordance with the terms applicable for US Federal Government officers or employees acting as part of their official duties; on a worldwide, perpetual, irrevocable, royalty-free basis to BMJ Publishing Group Ltd ("BMJ") its licensees and where the relevant Journal is co-owned by BMJ to the co-owners of the Journal, to publish the Work in this journal and any other BMJ products and to exploit all rights, as set out in our licence.

The Submitting Author accepts and understands that any supply made under these terms is made by BMJ to the Submitting Author unless you are acting as an employee on behalf of your employer or a postgraduate student of an affiliated institution which is paying any applicable article publishing charge ("APC") for Open Access articles. Where the Submitting Author wishes to make the Work available on an Open Access basis (and intends to pay the relevant APC), the terms of reuse of such Open Access shall be governed by a Creative Commons licence – details of these licences and which Creative Commons licence will apply to this Work are set out in our licence referred to above.

Other than as permitted in any relevant BMJ Author's Self Archiving Policies, I confirm this Work has not been accepted for publication elsewhere, is not being considered for publication elsewhere and does not duplicate material already published. I confirm all authors consent to publication of this Work and authorise the granting of this licence.

For peer review only

Title: A national administrative record linkage between specialist community drug and alcohol treatment data (The National Drug Treatment Monitoring System (NDTMS)) and inpatient hospitalisation data (Hospital Episode Statistics (HES)) in England; Design, Method and Evaluation

Authors:

Dr Emmert Roberts, National Addiction Centre and the Department of Psychological Medicine, Institute of Psychiatry, Psychology and Neuroscience, Kings College London, South London and the Maudsley NHS Foundation Trust and Public Health England

Dr James C. Doidge, Intensive Care National Audit & Research Centre

Dr Katie L. Harron, Great Ormond Street Institute of Child Health, University College London, London, UK

Professor Matthew Hotopf, Department of Psychological Medicine, Institute of Psychiatry, Psychology and Neuroscience, Kings College London and the South London and the Maudsley NHS Foundation Trust

Jonathan Knight, Public Health England

Martin White, Public Health England

Dr Brian Eastwood*, Public Health England

Professor Colin Drummond*, National Addiction Centre, Institute of Psychiatry, Psychology and Neuroscience, Kings College London and the South London and the Maudsley NHS Foundation Trust

* Both authors contributed equally

Corresponding author:

Dr Emmert Roberts
National Addiction Centre, Institute of Psychiatry, Psychology and Neuroscience, King's College London

For peer review only - http://bmjopen.bmj.com/site/about/guidelines.xhtml

+447754156145
emmert.roberts@kcl.ac.uk

The corresponding author has the right to grant on behalf of all authors and does grant on behalf of all authors, an exclusive licence (or non-exclusive for government employees) on a worldwide basis to the BMJ Publishing Group Ltd to permit this article (if accepted) to be published in BMJ editions and any other BMJPGL products and sublicences such use and exploit all subsidiary rights, as set out in our licence

Word Count: 3966 (Max 4000)

Competing Interest Statement:

All authors have completed the ICJME Unified Competing Interest form (available on request from the corresponding author) and declare: no support from any organisation for the submitted work; no financial relationships with organisations that might have an interest in the submitted work in the previous three years, no other relationships or activities that could appear to have influenced the submitted work.

Professor Hotopf is principal investigator of RADAR-CNS consortium - a public private partnership in collaboration with five pharma companies - Janssen, Biogen, UCB, MSD and Lundbeck, outside of the submitted work.

Transparency declaration:

The corresponding author (the manuscript's guarantor) affirms that the manuscript is an honest, accurate, and transparent account of the study being reported; that no important aspects of the study have been omitted; and that any discrepancies from the study as originally planned have been explained.

Role of the funding source:

This paper represents independent research funded by the Medical Research Council (MRC), as part of the corresponding author's MRC Addiction Research Clinical (MARC) Fellowship. The research was part funded by the NIHR Biomedical Research Centre at South London and Maudsley NHS Foundation Trust and King's College London, and by the NIHR Collaboration for Leadership in Applied Health Research and Care South London

For peer review only - http://bmjopen.bmj.com/site/about/guidelines.xhtml

(NIHR CLAHRC South London) now recommissioned as NIHR Applied Research Collaboration South London, and both CD and MH receive funding from an NIHR Senior Investigator award. The funders had no contribution to the study design; in the collection, analysis, and interpretation of data; in the writing of the report; and in the decision to submit the article for publication. All authors were independent from funders had full access to all of the data (including statistical reports and tables) in the study and take responsibility for the integrity of the data and the accuracy of the data analysis. The views expressed are those of the authors and not necessarily those of the MRC, the National Health Service (NHS), the NIHR, Public Health England (PHE) or the Department of Health and Social Care (DHSC).

For peer review only

Abstract (Word Count: 299; Max 300)

Objectives: The creation and evaluation of a national record linkage between substance misuse treatment, and inpatient hospitalisation data in England.

Design: A deterministic record linkage using personal identifiers to link the National Drug Treatment Monitoring System (NDTMS) curated by Public Health England (PHE), and Hospital Episode Statistics Admitted Patient Care (HES APC) curated by NHS Digital.

Setting and participants: Adults accessing substance misuse treatment in England between 1st April 2018 and 31st March 2019 (n=268,251) were linked to inpatient hospitalisation records available since 1st April 1997.

Outcome measures: Using a gold-standard subset, linked using NHS number, we report the overall linkage sensitivity and precision. Predictors for linkage error were identified, and inverse probability weighting was used to interrogate any potential impact on the analysis of length of hospital stay.

Results: 79.7% (n=213,814) people were linked to at least one HES record, with an estimated overall sensitivity of between 82.5% and 83.3%, and a precision of between 90.3% and 96.4%. Individuals were more likely to link if they were female, white, and aged between 46 and 60. Linked individuals were more likely to have an average length of hospital stay ≥ 5 days if they were male, older, had no fixed residential address, or had problematic opioid use. These associations did not change substantially after probability weighting, suggesting they were not affected by bias from linkage error.

Conclusions: Linkage between substance misuse treatment and hospitalisation records offers a powerful new tool to evaluate the impact of treatment on substance related harm in England. Whilst linkage error can produce misleading results, linkage bias appears to have little effect on the association between substance misuse treatment and length of hospital admission. As subsequent analyses are conducted, potential biases associated with the linkage process should be considered in the interpretation of any findings.

For peer review only - http://bmjopen.bmj.com/site/about/guidelines.xhtml

Strengths and limitations of this study

- This record linkage represents the first study of its kind to link centralised national level substance misuse treatment data and inpatient hospitalisation records
- No single unique identifier, such as NHS number, is routinely collected within NDTMS and fewer personal identifiers are routinely collected than in other UK government held datasets
- The limited availability of personal identifiers results in an increased risk of both false and missed matches, which could potentially affect the validity of any subsequently conducted analyses
- Linkage error did not appear to lead to systematic bias and misestimation of sociodemographic and clinical factor associations with the average length of hospital stay

For peer review only

Introduction

Routinely collected administrative data from the health and social care sector is increasingly used to both inform public health policy, and to generate research. Whilst several initiatives across the United Kingdom (UK) have used national record linkages to further population-level understanding in specific disease areas, (1) the ambition to harness record linkage as a means of improving health outcomes for people with drug and alcohol misuse has not been fully realised.

The number of people accessing specialist alcohol treatment has fallen by 19% between 2013 and 2017, while the number hospital admissions in which alcohol was recorded as a contributory factor has increased by 5% in the same timeframe. (2-4) Given this context, a recent report from the United Kingdom (UK) Department of Health and Social Care (DHSC) identified an urgent need to estimate the impact of specialist drug and alcohol treatment on acute care resource usage and substance-related harm. (5) The report posits that this goal may be achieved through detailed analysis of linked individual-level hospitalisation and substance misuse treatment data, which could '…*generate evidence to quantify the impact on health services utilisation before and after successful treatment*'. (5)

The National Drug Treatment Monitoring System (NDTMS) is the centralised database, collated and maintained by Public Health England (PHE), which receives monthly input from all local authority commissioned community drug and alcohol services in England. (6) This contains individual-level data on an individual's sociodemographic characteristics (date of birth, gender, ethnicity, housing status etc.), diagnostic characteristics, including the quantity and frequency of individuals' substance use, and treatment characteristics including frequency and type of contact with treatment services, the interventions received, and measures of treatment success. Hospital Episode Statistics (HES) is the centralised repository, collated and maintained by NHS Digital, which collects all information pertaining to National Health Service (NHS) hospitalisation in England and Wales. (7) The HES Admitted Patient Care (APC) database is one of the main administrative databases operating under the umbrella of HES and covers all NHS inpatient admissions, including any admission to private or third sector hospitals subsequently reimbursed by the NHS. (8) As such HES APC is estimated to contain > 99% of all inpatient hospital activity in England. (9) An inpatient hospital admission includes any secondary care-based activity requiring a hospital bed, thus includes day cases, and both planned and emergency admissions, in physical and mental health settings. HES APC does not cover accident and emergency

For peer review only - http://bmjopen.bmj.com/site/about/guidelines.xhtml

(A&E, emergency department) attendances, nor outpatient bookings, these data being held in separate HES databases.

Although NDTMS has been previously linked with mortality data from the Office of National Statistics (ONS), and the Police National Computer (PNC), (10-12) the lack of linkage between NDTMS and inpatient hospitalisation data limits the capacity to evaluate the impact of specialist drug and alcohol treatment on individual and regional rates of hospitalisation. International efforts have been made to facilitate record linkage of national databases in order to evaluate substance misuse outcomes, (13) however previous studies have often lacked access to national level data on substance misuse treatment, due in part to fragmented healthcare delivery systems, or lack of a centralised data repository. As centralised national databases exist for both hospitalisation and substance misuse treatment in England, we sought to link these two databases to inform drug and alcohol policy and research.

In this report we describe the process of record linkage and aim to evaluate the linkage quality and its potential impact on any subsequently conducted analyses. We believe this record linkage may result in the largest cross-sectional and longitudinal substance misuse database globally, and as such could become a resource which is able to support a large number of analytic outputs with the aim of improving the lives of those with substance use disorders.

For peer review only

Methods

Patients accessing publicly-funded specialist drug and alcohol treatment services in England provide written consent to share their information with NDTMS, and are informed that NDTMS records may be linked with data from specifically sanctioned UK government-held databases, including HES. (14) Over 98% of patients provide consent, (15) and the nature of this consent states that any record linkage would be undertaken by PHE, and that individuals may opt out at any time from having their records used within NDTMS. Approval to conduct the linkage was granted under regulation 3 of the Health Service (Control of Patient Information) Regulations 2002, (16) following review by the PHE Caldicott Advisory Panel (CAP) (Ref: CAP-2019-06).

Patient and Public Involvement

The study benefited throughout from discussion with the South London and the Maudsley (SLaM) Biomedical Research Centre (BRC) Data Linkage Service User and Carer Advisory Group, and the PHE Alcohol Treatment Expert Group which includes experts by experience.

Linkage methods

Record linkage is the process of bringing together information pertaining to the same individual (or entity) from different databases. Linkage applies a set of criteria to determine whether or not records belong to the same individual, and aims to assess the true match status of each record pair: either a ‘match’ i.e. records belong to the same individual, or a ‘non-match’ i.e. records belong to different individuals. If record pairs are misclassified, error may be introduced as either ‘false matches’ i.e. records from different individuals link erroneously, or ‘missed matches’ i.e. records from the same individual fail to link. Introduction of bias from linkage error, particularly if risk factors for important outcomes are associated with error rates, can impact the validity of findings derived from linked data. (17, 18) This is more likely to occur if datasets do not have a unique identifier in common. (19)

We selected all NDTMS records for adults accessing specialist drug or alcohol treatment in England between 1st April 2018 and 31st March 2019 as the test linkage population. The structure of the test linkage NDTMS data is such that one record represents one unique adult (n=268,251). (20) This was matched against all HES APC records available since database inception on 1st April 1997. The structure of the HES APC data is such that the same unique individual has multiple records, one for each hospital admission episode, with

For peer review only - http://bmjopen.bmj.com/site/about/guidelines.xhtml

unique individuals identified by a specific variable, the HESID, which is assigned by NHS Digital (n=390,642,220 records; n=67,378,943 unique individuals). (21) As not all individuals presenting to drug and alcohol services will have been admitted to hospital, we did not expect all NDTMS records to match with HES APC. No unique person identifiers, such as NHS number, were shared between both databases but a number of personal demographic and geographic identifiers were available for matching. Identifiers were harmonised to maintain a consistent format across the two databases, which included harmonising string length, use of spaces, capitalisation, and hyphens. Five variables were available for matching; an individual's date of birth (DOB), sex, postcode, ethnicity and GP practice. Full variable descriptions can be found in the online supplementary material.

A Structured Query Language (SQL) algorithm was designed to facilitate NDTMS to HES APC linkage. Initial data cleaning in both datasets included the conversion of all missing or non-valid data to null values and the collapse of all postcodes relating to a no fixed abode status (NFA) into a single value. All NDTMS records contained a validly coded value for DOB and sex whilst 96.3% had a validly coded postcode, 94.7% a validly coded ethnicity and 18.4% a validly coded GP practice. NDTMS records that had missing or invalid postcodes (n=10,011, 3.7%) were excluded from linkage, as a combination of sex, postcode and date of birth was the minimum - but not necessarily sufficient - data required to uniquely identify an individual. Of the remaining n=258,240 NDTMS records n=6,878 (2.7%) shared the same combination of dob, sex and postcode, of which n=164 (2.4%) did not have a valid entry for either ethnicity or GP practice.

Matching was based on an exact match for each of the five variables described above, and conducted hierarchically in four stages as below:

Stage 1: Exact match on dob, sex, postcode, ethnicity and GP practice
Stage 2: Exact match on dob, sex, postcode and GP practice
Stage 3: Exact match on dob, sex, postcode and ethnicity
Stage 4: Exact match on dob, sex and postcode

When records matched, they were removed from the dataset and not included in subsequent matching stages. As both databases are longitudinal, it was possible that several different values for postcode, and GP practice were recorded for each individual over time. Where more than one unique value was available the hierarchical algorithm attempted to link NDTMS records to HES APC records sequentially starting with the most

For peer review only - http://bmjopen.bmj.com/site/about/guidelines.xhtml

recent value for each variable. All resulting records that linked with multiple records from the other dataset were removed and treated as non-links.

Gold-standard subsample

A small subset of people in the full NDTMS sample (n=1,328), who to date were taking part in the PHE individual placement and support (IPS) trial, (22) had consented to make their unique ten-digit NHS number available. As NHS number is also coded within HES APC, this was used as a single unique identifier, common to both datasets, to facilitate linkage within this 'gold-standard' sample of individuals in NDTMS who had their NHS number available. The sociodemographic and clinical characteristics of the full NDTMS sample and the 'gold-standard' NDTMS sample are available in the online supplementary material as table S1.

Using the 'gold-standard' sample the linkage rate was calculated as the percentage of NDTMS individuals linked to any HES APC record first by exact matching on only NHS number, and secondly using the four-stage deterministic algorithm described above. The results were evaluated to determine the missed match rate, and the overall linkage precision, i.e. the proportion of links that are true.

Individuals linked using their NHS number were deemed to have been definitely hospitalised within their lifetime. Within this sample, individuals that were linked and not linked using the four-stage algorithm were compared to estimate rates of missed links. To allow for variation in patient characteristics and data quality between data providers, as well as between individuals, we used multilevel logistic regression with nesting of individuals within local authority commissioned treatment services and match status in NDTMS as the binary outcome (match=1, non-match=0). Model fit was examined using a likelihood ratio test comparing the multilevel model to a fixed-effects logistic model which did not account for nesting of individuals. We explored any association between match status and NDTMS sociodemographic (e.g. sex, age, ethnicity, NFA status, and Index of Multiple Deprivation (IMD)), and clinical factors (e.g. the misused substance/s for which the person entered treatment). For modelling purposes ethnicity was recoded into the binary categories of white and non-white, (23) and probability estimates of matching as a function of the independent variables were generated.

Analysis of linkage error

For peer review only - http://bmjopen.bmj.com/site/about/guidelines.xhtml

Challenges exist to assess the impact of linkage error when the outcome in question may not have been experienced by all people in the sample to be matched. When linking an individual's NDTMS records to HES APC it is difficult to know which matches have been missed as the HES database by design will only capture information about individuals who have been hospitalised. As such non-links could be due to an individual never having been admitted to hospital or being a missed match. (24, 25)

For each unique linked individual, a binary outcome of their average length of hospital stay ($\geq$ 5 days=1, < 5 days=0) was created to assess bias due to linkage error. This was chosen as it is clinically relevant, reflecting the current UK average length of hospital stay per person, and is recorded for all people within HES APC. (7) Using the estimated probability of matching from the 'gold-standard' analysis, we created a weight that was inversely proportional to the probability of being linked to HES APC data using the four-stage algorithm. These weights were subsequently assigned to each linked individual, as per standard methods to account for non-response bias in cross-sectional and cohort studies. (26, 27) Univariable multilevel logistic regression was used within the 'gold-standard' sample to examine the association between independent variables and the average length of hospital stay. Estimates were generated using the 'unbiased' linked sample matched using NHS number, and these were then compared to estimates obtained using the 'biased' linked sample matched using the four-stage algorithm. The model applied to the 'biased' sample was firstly conducted without any weighting, secondly conducted incorporating the inverse probability weights to examine if this corrected any linkage error, and thirdly conducted weighted according to the odds of having sufficient matching data.

Data Access

Whilst access to the linked dataset is only available within PHE, subject to approval, extracts of NDTMS are available to researchers through the Office of Data Release (ODR) at PHE, (28) and extracts of HES APC are available through the Data Access Request Service (DARS) at NHS Digital. (29) In addition, the code for our linkage algorithm will also be made available upon request to PHE.

The linkage was conducted using SQL Server Management Studio version 18.4. Additional analyses were conducted using STATA MP version 15.1, with the significance level set at 0.05.

For peer review only - http://bmjopen.bmj.com/site/about/guidelines.xhtml

Results

The overall matching for a unique person within the full NDTMS sample (n=268,251) to a HES APC hospitalisation record generated n=213,814 linked records, representing a linkage rate of 79.7%. The proportion linked according to the matching stages described above were: stage one: 10.7%, stage two: 5.7%, stage three: 72.5%, and stage four: 11.1%.

Gold-standard subsample

The overall matching for a unique person within NDTMS to a HES APC hospitalisation record using the 'gold-standard' subset of people with an NHS number available in NDTMS, generated n=1,153 linked records using NHS number, representing a linkage rate of 86.6%. Using the four-stage algorithm within the 'gold-standard' population generated n=1,053 linked records with a linkage rate of 79.3%. Although this was lower than the NHS number match rate this suggests that the majority of unlinked records were true non-links (i.e. individuals who had not previously been hospitalised) and not missed matches. Of the n=1,053 records linked using the four-stage algorithm, 102 were not linked by the gold standard. These included n=36 records that disagreed on NHS number and were therefore assumed to be false links, and 66 that had missing or invalid NHS numbers and could represent either false links or links missed by the gold standard. These two possibilities suggest a precision of between 90.3% and 96.4%, respectively. Of the n=1,153 records matched using the NHS number n=202 were not matched by the four-stage algorithm suggesting a sensitivity of between 82.5% and 83.3%, respectively.

Table one summarises the associations between sociodemographic and clinical variables and linkage by four-stage algorithm within the gold-standard subsample who linked to HES APC via their NHS number (n=1,153). Within this sample we compared individuals who were classified as linked or non-linked using the four-stage algorithm, an adjusted odds ratio (aOR) greater than 1 denoting increased odds of successful linkage when compared to the reference value. In the adjusted model, we found significant differences in the odds of linking for sex, age and ethnicity. There was strong evidence that when compared with women, men were significantly less likely to link to HES APC (aOR 0.48, 95%CI 0.30 to 0.79, p=0.003), when compared to those aged between 18-30, those aged between 46-60 were significantly more likely to link (aOR 2.28, 95%CI 1.08 to 4.82, p=0.03), and when compared to people of a white ethnicity, people with a non-white ethnicity were significantly less likely to link (aOR 0.35, 95%CI 0.20 to 0.63, p<0.001). The multilevel model was significantly

For peer review only - http://bmjopen.bmj.com/site/about/guidelines.xhtml

superior to the fixed-effects logistic model ($p<0.001$), with an intraclass correlation coefficient (ICC) of 0.13 (95%CI 0.04 to 0.36).

Analysis of linkage error

Weighting the probability of being linked to HES APC data using the four-stage algorithm demonstrated a correction of linkage bias within the 'gold-standard' sample, the results of which are summarised in table S2 in the online supplementary material.

The full linked sample had a total of 1,624,152 inpatient hospital admissions since HES database inception in April 1997 until January 2020, with a total time spent in hospital of 14,461 years, and an overall average length of hospital stay of three days. Table two summarises the associations between sociodemographic and clinical variables and the average length of hospital admission for linked individuals differentiated into those with an average length of hospital admission < 5 days and those with an average length of hospital admission ≥ 5 days. An adjusted odds ratio (aOR) greater than 1 denotes increased odds of an average length of hospital admission ≥ 5 days when compared to the reference value. In the adjusted model, we found significant differences in the average length of hospital admission across the majority of studied sociodemographic and clinical factors. There were no substantial differences between the estimates generated from the adjusted models following inverse probability or sufficient matching data weighting. The multilevel model was significantly superior to the fixed-effects logistic model ($p<0.001$), with an ICC of 0.02 (95%CI 0.01 to 0.02).

For peer review only

Discussion

Using deterministic matching, a national longitudinal and cross-sectional dataset was built between NDTMS specialist community drug and alcohol treatment data and HES hospitalisation data in England, providing a linkage for 213,814 adults (79.7% of the full NDTMS cohort) to their inpatient hospital records. Using our linkage algorithm there were significant differences in the sociodemographic and clinical characteristics between the linked and non-linked samples, with individuals more likely to link if they were female, white, and aged between 46 and 60 years old. Using the linked data, we were able to demonstrate that individuals were more likely to have an increased average length of hospital stay if they were male, older, had no fixed residential address, and had problematic opioid use. These effects did not change substantially following inverse probability weighting, suggesting they were not driven by bias from linkage error.

Analysis of Linkage Biases

Very few studies have examined linkage error in the context of people with substance use disorders. Using our deterministic algorithm, n=54,437 (20.3%) of individuals were not linked to HES APC hospitalisation records. Linkage of the gold-standard sample suggests that approximately two thirds of these are true non-links (i.e. arising because the individual had never been hospitalised and therefore had no HES record), and the remaining third are missed matches. When using the 'gold-standard' sample 86.6% of records matched using NHS number, as such 86.6% is likely to estimate the overall true match rate. We can thus infer that a roughly similar percentage of the n=10,011 NDTMS records with insufficient matching data should match and are therefore genuine missed-matches in the total sample (n=8,670). Based on our linkage sensitivity these 8,670 records constitute just under half of the total number of likely missed match records when using the four-stage algorithm. The sociodemographic and clinical characteristics of this cohort can be found in the online supplementary material as table S3, and when compared to the NDTMS cohort with sufficient matching data, demonstrate a substantially lower odds of having sufficient matching data if individuals were male, younger, and problematic opioid users. This indicates a higher likelihood of missed matches within these groups which is in accordance with the reduced odds of linkage for male, younger and non-white individuals observed using the four-stage algorithm in the 'gold-standard' sample.

We found that older age groups were more likely to link which may reflect a greater availability of accurate personal identifiers in the records of this population as by living longer

For peer review only - http://bmjopen.bmj.com/site/about/guidelines.xhtml

they have had greater potential exposure to drug and alcohol services compared to other age groups, and an increased number of hospitalisation records, and therefore potentially more values of matching variables. Previous research has suggested that individuals from black and ethnic minorities are more likely to have administrative records with inaccurately recorded dates of birth and higher levels of residential instability, which may be applicable to this sample, and partially account for the reduced likelihood in of linkage compared to white individuals. (30) It is reassuring however that in our sample, linkage biases do not appear to have significant effect on the associations between substance misuse and average length of hospital stay.

Strengths and limitations of the matching methods and evaluation

This represents the first study of its kind to link centralised national level substance misuse treatment data and inpatient hospitalisation records, and provides an example of how potential non-random loss between routinely collected administrative datasets can be adjusted for by weighting techniques. (31) As we had access to complete source data records, we were able to demonstrate that linkage error did not appear to lead to systematic bias and misestimation of sociodemographic and clinical factor associations with average length of hospital stay. It should be noted that in order to evaluate potential linkage bias within this paper we only report a single healthcare outcome. Following evaluation for potential linkage bias interrogation of the resultant dataset will be possible to address a number of key research and policy questions. There are also a number of limitations. Due to the previous practice of the UK Home Office compiling full names and addresses of all registered addicts in its 'Index of Addicts', (32) and more generally the stigma experienced by people with substance use disorders, NDTMS is careful to collect only the minimum amount of personal identifier information it deems necessary to balance the need for population surveillance, with legitimate concerns about individual identification. An unfortunate consequence, however, is that no single unique identifier, such as NHS number, is routinely collected within NDTMS and the personal identifiers which are collected are typically fewer than in other UK government held datasets. This creates a unique problem for NDTMS data linkage, which is compounded by the fact that, when compared to the general population, individuals within NDTMS are also less likely to be registered with a GP, more likely to not have a residential address, and potentially have an interest in providing non-accurate personal identification information to drug and alcohol services. All of the above reasons may contribute to the observed increased rate of false and missed matches compared to other national data linkages. (31) Nevertheless, a national centralised data repository for substance misuse treatment presents a unique opportunity to link with other

For peer review only - http://bmjopen.bmj.com/site/about/guidelines.xhtml

For peer review only

health and social care record systems, provided consent is given by those individuals, in an attempt to improve the lives of people with substance use disorders. This paucity of available personal identifiers results in an increased risk of both false and missed matches, particularly at lower confidence matching stages and these limitations could have led to our match rate being an overestimation of the linkage performance. As such in order to minimise the risk of false matches, records that linked with multiple different unique records from either dataset were removed and treated as non-links. These could reflect imperfect internal linkage or deduplication of NDTMS or HES; i.e. these could be true multiple links and this linkage strategy could increase the rate of missed matches. When 'gold-standard' data are used to assess linkage quality this is assumed to be representative in terms of the distribution of the quality of matching and analysis variables. Although our 'gold-standard' dataset unique person identifier is NHS number we cannot exclude the possibility there may be coding errors within NHS numbers and the dataset may not represent the remainder of records. Although there was a significantly lower linkage rate using our algorithm compared to using NHS number within the gold-standard sample (79.7% vs 86.6%), differences in ethnicity and age between the full and gold-standard NDTMS samples partially explain this difference, but do not appear to contribute significant bias due to linkage error.

Implications

This linkage between substance misuse treatment and hospitalisation records offers a new powerful tool to evaluate the impact of specialist treatment on alcohol and drug related harm in England. Through its interrogation, and via additional sanctioned linkage to datasets from other government departments, e.g. the Department for Work and Pensions (DWP), this data may hopefully be able to provide insight and knowledge to improve the lives of people with substance use disorders. Whilst biases due to linkage error may produce misleading results in our sample, linkage biases appear to have little effect on the association between drug and alcohol treatment and length of hospital admission. However, without ongoing ability to probe information within the source data, potential linkage error could be introduced without future analysts being aware that there was need for it to be accounted for.

In time, we hope this resource will generate a wide network of granular data and analytical expertise, which can be used to inform both commissioning and service provision to better meet the needs of people with substance use disorders in England. The immediate next steps are to evaluate the most common reasons for hospital admission within the cohort of people accessing drug and alcohol treatment and to assess the impact of engagement in, and successful completion of, drug and alcohol treatment on individual and national rates

For peer review only

hospitalisation. It is important to note that as subsequent analyses of the resultant linked dataset are conducted, any potential bias associated with the linkage process should always be considered in the interpretation of any findings.

For peer review only

References

1. Enabling Data Linkage to Maximise the Value of Public Health Research Data: full report https://wellcome.ac.uk/sites/default/files/enabling-data-linkage-to-maximise-value-of-public-health-research-data-phrdf-mar15.pdf.
2. Statistics on Alcohol England 2018 https://files.digital.nhs.uk/60/B4D319/alc-eng-2018-rep.pdf.
3. Adult substance misuse statistics rom the National Drug Treatment Monitoring System (NDTMS) 1 April 2017 to 31 March 2018 https://assets.publishing.service.gov.uk/government/uploads/system/uploads/attachment_data/file/752993/AdultSubstanceMisuseStatisticsfromNDTMS2017-18.pdf.
4. PHE inquiry into the fall in numbers of people in alcohol treatment: findings Published https://www.gov.uk/government/publications/alcohol-treatment-inquiry-summary-of-findings/phe-inquiry-into-the-fall-in-numbers-of-people-in-alcohol-treatment-findings#the-inquiry-process. 2018.
5. Hill-McManus D, Stone T, Ally A, Pryce RE, Gillespie D, Buykx P, et al. An Evidence-Based Model for Estimating Requirements for Specialist Alcohol Treatment Capacity in England The Specialist Treatment for Alcohol Model (STreAM) Version 1.0. 2016.
6. National Drug Treatment Monitoring System https://digital.nhs.uk/data-and-information/information-standards/information-standards-and-data-collections-including-extractions/publications-and-notifications/standards-and-collections/dcb0107-national-drug-treatment-monitoring-system.
7. Hospital Admitted Patient Care Activity https://digital.nhs.uk/data-and-information/publications/statistical/hospital-admitted-patient-care-activity.
8. Herbert A, Wijlaars L, Zylbersztejn A, Cromwell D, Hardelid P. Data resource profile: hospital episode statistics admitted patient care (HES APC). International journal of epidemiology. 2017;46(4):1093-i.
9. Healthcare across the UK: A comparison of the NHS in England, Scotland, Wales and Northern Ireland https://www.nao.org.uk/wp-content/uploads/2012/06/1213192.pdf.
10. Willey H, Eastwood B, Gee IL, Marsden J. Is treatment for alcohol use disorder associated with reductions in criminal offending? A national data linkage cohort study in England. Drug and alcohol dependence. 2016;161:67-76.
11. White M, Burton R, Darke S, Eastwood B, Knight J, Millar T, et al. Fatal opioid poisoning: a counterfactual model to estimate the preventive effect of treatment for opioid use disorder in England. Addiction. 2015;110(8):1321-9.
12. Drug and alcohol addiction, and obesity: effects on employment outcomes https://www.gov.uk/government/publications/drug-and-alcohol-addiction-and-obesity-effects-on-employment-outcomes.
13. Peacock A, Chiu V, Leung J, Dobbins T, Larney S, Gisev N, et al. Protocol for the Data-Linkage Alcohol Cohort Study (DACS): investigating mortality, morbidity and offending among people with an alcohol-related problem using linked administrative data. BMJ Open. 2019;9(8):e030605.
14. NDTMS: consent and confidentiality guidelines https://www.gov.uk/government/publications/confidentiality-guidance-for-drug-and-alcohol-treatment-providers-and-clients.

For peer review only - http://bmjopen.bmj.com/site/about/guidelines.xhtml

15. Marsden J, Eastwood B, Bradbury C, Dale-Perera A, Farrell M, Hammond P, et al. Effectiveness of community treatments for heroin and crack cocaine addiction in England: a prospective, in-treatment cohort study. The Lancet. 2009;374(9697):1262-70.

16. The Health Service (Control of Patient Information) Regulations 2002 http://www.legislation.gov.uk/uksi/2002/1438/contents/made.

17. Lariscy JT. Differential record linkage by Hispanic ethnicity and age in linked mortality studies: implications for the epidemiologic paradox. Journal of aging and health. 2011;23(8):1263-84.

18. Doidge JC, Harron KL. Reflections on modern methods: linkage error bias. International Journal of Epidemiology. 2019.

19. Harron K, Goldstein H, Dibben C. Methodological developments in data linkage: John Wiley & Sons; 2015.

20. Substance misuse treatment for adults: statistics 2018 to 2019 https://www.gov.uk/government/statistics/substance-misuse-treatment-for-adults-statistics-2018-to-2019.

21. Replacement of the HES Patient ID (HESID) https://www.cl.cam.ac.uk/~rja14/Papers/HESID_Replacement_Nov09.pdf.

22. Marsden J, Anders P, Clark H, Colocassis K, Eastwood B, Knight J, et al. Protocol for a multi-centre, definitive randomised controlled trial of the effectiveness of Individual Placement and Support for employment support among people with alcohol and drug dependence. Trials. 2020;21(1):1-12.

23. Marsden J, Eastwood B, Jones H, Bradbury C, Hickman M, Knight J, et al. Risk adjustment of heroin treatment outcomes for comparative performance assessment in E ngland. Addiction. 2012;107(12):2161-72.

24. Bohensky MA, Jolley D, Sundararajan V, Evans S, Pilcher DV, Scott I, et al. Data linkage: a powerful research tool with potential problems. BMC health services research. 2010;10(1):346.

25. Harron KL, Doidge JC, Knight HE, Gilbert RE, Goldstein H, Cromwell DA, et al. A guide to evaluating linkage quality for the analysis of linked data. International journal of epidemiology. 2017;46(5):1699-710.

26. Höfler M, Pfister H, Lieb R, Wittchen H-U. The use of weights to account for non-response and drop-out. Social psychiatry and psychiatric epidemiology. 2005;40(4):291-9.

27. Little RJ, Vartivarian S. On weighting the rates in non-response weights. Statistics in medicine. 2003;22(9):1589-99.

28. Accessing PHE data through the Office for Data Release https://www.gov.uk/government/publications/accessing-public-health-england-data/about-the-phe-odr-and-accessing-data.

29. Data Access Request Service (DARS) https://digital.nhs.uk/services/data-access-request-service-dars.

30. Hagger-Johnson G, Harron K, Goldstein H, Aldridge R, Gilbert R. Probabilistic linking to enhance deterministic algorithms and reduce linkage errors in hospital administrative data. Journal of innovation in health informatics. 2017;24(2):891.

31. Downs JM, Ford T, Stewart R, Epstein S, Shetty H, Little R, et al. An approach to linking education, social care and electronic health records for children and young people in South London: a linkage study of child and adolescent mental health service data. BMJ open. 2019;9(1):e024355.

For peer review only

32. Ghodse AH, Sheehan M, Taylor C, Edwards G. Deaths of drug addicts in the United Kingdom 1967-81. Br Med J (Clin Res Ed). 1985;290(6466):425-8.

For peer review only

Tables

Table one: Sociodemographic and clinical characteristics of the n=1,153 individuals in NDTMS linked to HES APC using NHS number characterised as either linked or non-linked to HES APC using the four-stage algorithm

		Linked Pairs n (%)	Non-Linked Residuals n (%)	OR (95% CI) for positive linkage	p value	aOR[1] (95%CI) for positive linkage	p value
**All**	**All**	951 (82.5)	202 (17.5)	-	-	-	-
**Sociodemographic**	**Sex**						
	Female	320 (86.5)	50 (13.5)	Reference		Reference	
	Male	631 (80.6)	152 (19.4)	0.66 (0.46-0.95)	0.03*	0.48 (0.30-0.79)	0.003*
	**Age in years (at presentation to D&A services)**						
	18-30	97 (81.5)	22 (18.5)	Reference		Reference	
	31-45	497 (79.1)	131 (20.9)	0.79 (0.46-1.36)	0.40	1.10 (0.56-2.19)	0.78
	46-60	336 (87.7)	47 (12.3)	1.53 (0.85-2.76)	0.16	2.28 (1.08-4.82)	0.03*
	60+	21 (91.3)	2 (8.7)	1.62 (0.34-7.83)	0.55	1.79 (0.33-9.62)	0.50
	**Deprivation (IMD) Quintile**						
	First (Most deprived)	328 (81.6)	74 (18.4)	Reference		Reference	
	Second	275 (85.1)	48 (14.9)	0.96 (0.62-1.49)	0.87	0.81 (0.50-1.32)	0.40
	Third	210 (90.5)	22 (9.5)	1.38 (0.77-2.47)	0.28	1.04 (0.55-1.98)	0.90
	Fourth	89 (89.9)	10 (10.1)	1.07 (0.49-2.34)	0.87	1.22 (0.46-3.23)	0.69
	Fifth (Least deprived)	17 (85.0)	3 (15.0)	1.14 (0.30-4.30)	0.85	0.49 (0.12-1.99)	0.32
	**Residential Status[2]**						
	Non NFA postcode	913 (81.9)	202 (18.1)	Reference		Reference	
	NFA postcode	38 (100.0)	0 (0.0)	-	-	-	-
	**Ethnicity[3]**						
	White	840 (88.9)	105 (11.1)	Reference		Reference	
	Non-white	80 (73.4)	29 (26.6)	0.42 (0.24-0.71)	<0.001*	0.35 (0.20-0.63)	<0.001*
**Clinical**	**Substance Misuse[4]**						
	Opioid	503 (77.3)	148 (22.7)	Reference		Reference	
	Alcohol only	311 (90.7)	32 (9.3)	2.41 (1.57-3.68)	<0.001*	1.57 (0.95-2.61)	0.08
	Non-opioid and alcohol	75 (91.5)	7 (8.5)	2.16 (0.95-0.4.93)	0.07	2.03 (0.76-5.44)	0.16
	Non-opioid only	62 (80.5)	15 (19.5)	1.12 (0.59-2.12)	0.73	0.98 (0.44-2.18)	0.96

* p<0.05; OR Odds Ratio; aOR Adjusted Odds Ratio; CI Confidence Interval; IMD Index of Multiple Deprivation

1 Adjusted for all other covariates listed in table; 2 Residential status was omitted from the model as all people with an NFA postcode were linked 3 Office of Population Censuses and Surveys (OPCS) categories A, B and C collapsed as white, all other OPCS categories (D-S) collapsed as non-white; 4 NDTMS categorisation **opioid**: clients with any mention of opioid use in any treatment episode during the year irrespective of other substances cited; **alcohol only:** clients who present with problems related to alcohol but no other substances; **non-opioid and alcohol:** clients with non-opioid drug *and* alcohol use problems (but *not* opioids) recorded in any treatment episode during the year; **non-opioid only:** clients who present for treatment related to non-opioid drug use but *not* opioids or alcohol

For peer review only

Table two: The odds of an average ≥ 5 day length of hospital admission in the 213,814 people in treatment at drug and alcohol services

		≥ 5 days average hospital admission length n (%)	< 5 days average hospital admission length n (%)	OR (95%CI)	aOR[1] (95%CI)	Inverse probability weighted (IPW) for missed links aOR[2] (95%CI)	Weighted for sufficient matching data aOR (95% CI)
**All**	**All**	25,814 (12.1)	188,000 (87.9)				
**Sociodemographic**	**Sex**						
	Female	6,715 (9.4)	65,114 (90.6)	Reference	Reference	Reference	Reference
	Male	19,099 (13.5)	122,886 (86.5)	1.51 (1.47-1.56)	1.42 (1.37-1.46)	1.42 (1.37-1.47)	1.42 (1.37-1.47)
	**Age in years (at presentation to D&A services)**						
	18-30	1,979 (7.1)	26,040 (92.9)	Reference	Reference	Reference	Reference
	31-45	10,804 (10.9)	88,392 (89.1)	1.58 (1.50-1.66)	1.46 (1.38-1.54)	1.46 (1.37-1.54)	1.46 (1.37-1.54)
	46-60	10,771 (14.7)	62,634 (85.3)	2.17 (2.06-2.28)	2.08 (1.96-2.20)	2.07 (1.95-2.20)	2.08 (1.96-2.20)
	60+	2,260 (17.1)	10,934 (82.9)	2.62 (2.46-2.80)	2.84 (2.65-3.06)	2.81 (2.61-3.04)	2.84 (2.63-3.06)
	**Deprivation (IMD) Quintile**						
	First (Most deprived)	8,340 (12.4)	58,831 (87.6)	Reference	Reference	Reference	Reference
	Second	6,988 (12.4)	49,543 (87.6)	0.99 (0.95-1.03)	1.01 (0.97-1.05)	1.01 (0.97-1.06)	1.01 (0.97-1.06)
	Third	4,560 (11.5)	35,324 (88.5)	0.94 (0.90-0.98)	0.98 (0.94-1.02)	0.98 (0.93-1.04)	0.98 (0.93-1.04)
	Fourth	3,045 (10.8)	25,250 (89.2)	0.90 (0.86-0.95)	0.95 (0.90-1.00)	0.95 (0.90-1.01)	0.95 (0.90-1.01)
	Fifth (Least deprived)	1,289 (10.0)	11,599 (90.0)	0.85 (0.79-0.91)	0.90 (0.84-0.97)	0.90 (0.84-0.97)	0.90 (0.85-0.97)
	**Residential Status**						
	Non NFA postcode	24,108 (11.8)	180,389 (88.2)	Reference	Reference	Reference	Reference
	NFA postcode	1,706 (18.3)	7,611 (81.7)	1.65 (1.57-1.75)	1.44 (1.27-1.63)	1.43 (1.24-1.65)	1.43 (1.24-1.65)
	**Ethnicity[3]**						
	White	22,808 (11.8)	170,772 (88.2)	Reference	Reference	Reference	Reference
	Non-white	2,462 (15.3)	13,650 (84.7)	1.20 (1.14-1.26)	1.19 (1.13-1.25)	1.18 (1.11-1.26)	1.19 (1.12-1.27)
	**Substance Misuse[4]**						
**Clinical**	Opioid	15,309 (14.3)	92,102 (85.7)	Reference	Reference	Reference	Reference
	Alcohol only	6,776 (10.4)	58,084 (89.6)	0.71 (0.69-0.74)	0.68 (0.66-0.70)	0.68 (0.65-0.72)	0.68 (0.64-0.71)
	Non-opioid and alcohol	2,055 (9.0)	20,820 (91.0)	0.58 (0.55-0.61)	0.67 (0.63-0.70)	0.67 (0.63-0.72)	0.67 (0.62-0.71)
	Non-opioid only	1,674 (9.0)	16,994 (91.0)	0.59 (0.56-0.62)	0.74 (0.70-0.79)	0.76 (0.70-0.82)	0.74 (0.69-0.80)

OR Odds Ratio; aOR Adjusted Odds Ratio; CI Confidence Interval; IMD Index of Multiple Deprivation

1 Adjusted for all other covariates listed in table; 2 Adjusted model with inverse probability weighting for matching included; 3 Office of Population Censuses and Surveys (OPCS) categories A, B and C collapsed as white, all other OPCS categories (D-S) collapsed as non-white; 4 NDTMS categorisation **opioid**: clients with any mention of opioid use in any treatment episode during the year irrespective of other substances cited; **alcohol only:** clients who present with problems related to alcohol but no other substances; **non-opioid and alcohol:** clients with non-opioid drug *and* alcohol use problems (but *not* opioids) recorded in any treatment episode during the year; **non-opioid only:** clients who present for treatment related to non-opioid drug use but *not* opioids or alcohol

For peer review only

For peer review only - http://bmjopen.bmj.com/site/about/guidelines.xhtml

Online Supplementary Material

Contents

Tables

S1: Differences between full NDTMS cohort and the 'gold-standard' NDTMS cohort with NHS number available

S2: The odds of an average ≥ 5 day length of hospital admission within the 'gold-standard' NDTMS cohort with NHS number available, in the 'unbiased' sample matched using NHS number and the 'biased' sample matched using the four-stage algorithm, following application of inverse probability weighting to correct for potential linkage bias

S3: Differences between NDTMS cohort that contains sufficient matching data (i.e. a validly coded dob, sex and postcode) and the NDTMS cohort that does not contain sufficient matching data

S4: Description of variables available for linkage

References

For peer review only

Tables

Table S1: Differences between full NDTMS cohort and the 'gold-standard' NDTMS cohort with NHS number available

		Full NDTMS Cohort n (%)	Cohort with NHS number n (%)	p value
**All**		268,251 (100.0)	1,328 (100.0)	
**Sociodemographic**	**Sex**			
	Female	83,015 (31.0)	393 (29.6)	0.29
	Male	185,236 (69.0)	935 (70.4)	
	**Age in years (at presentation to D&A services)**			
	18-30	36,068 (13.4)	143 (10.8)	0.04*
	31-45	127,635 (47.6)	720 (54.2)	
	46-60	89,643 (33.4)	438 (33.0)	
	60+	14,905 (5.6)	27 (2.0)	
	**Deprivation (IMD) Quintile**			
	First (Most deprived)	81,478 (33.2)	472 (39.9)	<0.001*
	Second	68,808 (28.0)	366 (29.4)	
	Third	47,139 (19.2)	271 (21.8)	
	Fourth	33,354 (13.6)	111 (8.9)	
	Fifth (Least deprived)	15,002 (6.1)	25 (2.0)	
	**Residential Status**			
	Non NFA postcode	255,515 (95.3)	1,286 (96.8)	0.01*
	NFA postcode	12,736 (4.7)	42 (3.2)	
	**Ethnicity**[1]			
	White	230,012 (90.6)	1,064 (87.5)	<0.001*
	Non-white	23,928 (9.4)	152 (12.5)	
**Clinical**	**Substance Misuse**[2]			
	Opioid	139,845 (52.1)	775 (58.4)	<0.001*
	Alcohol only	75,555 (28.2)	362 (27.3)	
	Non-opioid and alcohol	28,598 (10.7)	95 (7.1)	
	Non-opioid only	24,253 (9.0)	96 (7.2)	

* p<0.05; 1 Office of Population Censuses and Surveys (OPCS) categories A, B and C collapsed as white, all other OPCS categories (D-S) collapsed as non-white
2 NDTMS categorisation **opioid**: clients with any mention of opioid use in any treatment episode during the year irrespective of other substances cited; **alcohol only:** clients who present with problems related to alcohol but no other substances; **non-opioid and alcohol:** clients with non-opioid drug *and* alcohol use problems (but *not* opioids) recorded in any treatment episode during the year; **non-opioid only:** clients who present for treatment related to non-opioid drug use but *not* opioids or alcohol

Table S2: The conditional odds of an average ≥ 5 day length of hospital admission within the 'gold-standard' NDTMS cohort with NHS number available, in the 'unbiased' sample matched using NHS number and the 'biased' sample matched using the four-stage algorithm, following application of inverse probability weighting to correct for potential linkage bias

		'Unbiased' sample matched using NHS number		'Biased' sample matched using four-stage algorithm		
		n (%)	OR (95%CI)	n (%)	OR (95%CI)	Weighted OR[1] (95%CI)
	**All**	1,153 (100.0)	-	1,053 (100.0)	-	-
**Sociodemographic**	**Sex**					
	Female	370 (32.1)	Reference	336 (31.9)	Reference	Reference
	Male	783 (67.9)	1.27 (0.80-2.02)	717 (68.1)	1.33 (0.85-2.09)	1.26 (0.80-2.00)
	**Age in years (at presentation to D&A services)**					
	≤ 45	747 (64.8)	Reference	658 (62.5)	Reference	Reference
	> 45	406 (35.2)	1.07 (0.69-1.65)	395 (37.5)	1.17 (0.78-1.77)	1.11 (0.71-1.73)
	**Deprivation (IMD) Quintile**					
	First (Most deprived)	402 (37.4)	Reference	363 (35.7)	Reference	Reference
	All other quintiles	674 (62.6)	0.64 (0.42-1.00)	655 (64.3)	0.71 (0.47-1.08)	0.70 (0.40-1.10)
	**Residential Status**					
	Non NFA postcode	1,115 (96.7)	Reference	1,011 (96.0)	Reference	Reference
	NFA postcode	38 (3.3)	0.59 (0.13-2.51)	42 (4.0)	0.56 (0.13-2.35)	0.60 (0.12-2.49)
	**Ethnicity**[2]					
	White	945 (89.7)	Reference	915 (90.0)	Reference	Reference
	Non-white	109 (10.3)	1.74 (0.90-3.36)	102 (10.0)	2.19 (1.25-3.84)	1.89 (0.99-3.39)
**Clinical**	**Substance Misuse**[3]					
	Opioid	651 (56.5)	Reference	569 (54.0)	Reference	Reference
	All other drug categories	502 (43.5)	0.67 (0.43-1.04)	484 (46.0)	0.65 (0.43-0.99)	0.65 (0.42-1.00)

OR Odds Ratio; CI Confidence Interval; IMD Index of Multiple Deprivation

1 Model with inverse probability weighting for matching included; 2 Office of Population Censuses and Surveys (OPCS) categories A, B and C collapsed as white, all other OPCS categories (D-S) collapsed as non-white; 3 NDTMS categorisation **opioid**: clients with any mention of opioid use in any treatment episode during the year irrespective of other substances cited; **alcohol only:** clients who present with problems related to alcohol but no other substances; **non-opioid and alcohol:** clients with non-opioid drug *and* alcohol use problems (but *not* opioids) recorded in any treatment episode during the year; **non-opioid only:** clients who present for treatment related to non-opioid drug use but *not* opioids or alcohol

For peer review only

Table S3: Differences between NDTMS cohort that contains sufficient matching data (i.e. a validly coded dob, sex and postcode) and the NDTMS cohort that does not contain sufficient matching data

		NDTMS cohort that does contain sufficient matching data n (%)	NDTMS cohort that does not contain sufficient matching data n (%)	OR (95%CI)	aOR (95%CI)[1]
**All**		258,240 (100.0)	10,011 (100.0)	-	
**Sociodemographic**	**Sex**				
	Female	80,111 (31.0)	2,904 (29.0)	Reference	Reference
	Male	178,129 (69.0)	7,107 (71.0)	1.10 (1.05-1.15)	1.27 (1.15-1.40)
	**Age in years (at presentation to D&A services)**				
	18-30	35,446 (13.7)	622 (6.2)	Reference	Reference
	31-45	122,999 (47.6)	4,636 (56.3)	2.15 (1.97-2.34)	0.94 (0.83-1.06)
	46-60	85,425 (33.1)	4,218 (42.1)	2.81 (2.58-3.06)	0.61 (0.53-0.71)
	60+	14,370 (5.6)	535 (5.3)	2.12 (1.89-2.39)	0.52 (0.40-0.67)
	**Deprivation (IMD) Quintile[2]**				
	First (Most deprived)	81,478 (33.2)	-	-	-
	Second	68,808 (28.0)	-	-	-
	Third	47,139 (19.2)	-	-	-
	Fourth	33,354 (13.6)	-	-	-
	Fifth (Least deprived)	15,002 (6.1)	-	-	-
	**Residential Status[2]**				-
	Non NFA postcode	245,504 (95.1)	-	-	-
	NFA postcode	12,736 (4.9)	-	-	-
	**Ethnicity[3]**				
	White	228,023 (90.6)	1,989 (89.2)	Reference	Reference
	Non-white	23,688 (9.4)	240 (10.8)	1.16 (1.02-1.33)	0.84 (0.73-0.97)
**Clinical**	**Substance Misuse[4]**				
	Opioid	131,316 (50.9)	8,529 (85.2)	Reference	Reference
	Alcohol only	74,840 (29.0)	715 (7.1)	0.15 (0.14-0.16)	0.86 (0.77-0.95)
	Non-opioid and alcohol	28,202 (10.9)	396 (4.0)	0.22 (0.20-0.24)	1.07 (0.93-1.22)
	Non-opioid only	23,882 (9.3)	371 (3.7)	0.24 (0.22-0.27)	1.05 (0.91-1.22)

1 Adjusted for all other covariates listed in table 2 All records that did not contain sufficient matching data lacked validly coded postcodes, as such no IMD quintile or residential status values were available for this cohort. 3 Office of Population Censuses and Surveys (OPCS) categories A, B and C collapsed as white, all other OPCS categories (D-S) collapsed as non-white 4 NDTMS categorisation **opioid**: clients with any mention of opioid use in any treatment episode during the year irrespective of other substances cited; **alcohol only:** clients who present with problems related to alcohol but no other substances; **non-opioid and alcohol:** clients with non-opioid drug *and* alcohol use problems (but *not* opioids) recorded in any treatment episode during the year; **non-opioid only:** clients who present for treatment related to non-opioid drug use but *not* opioids or alcohol

For peer review only

Table S4: Description of variables available for linkage

Variable	Format	Notes
Date of Birth (*dob*)	'yyyymmdd'	
Sex (*sex*)	1=male; 2=female	
Sector Level Postcode (*postcode*)	e.g. 'S752' or 'NE177'	This contains the entire 'outcode' and the first character of the 'incode', each unique sector level postcode representing roughly 3000 households in England (1)
Ethnicity (*ethnicity*)	e.g. 'A'	16 categories A - S harmonised to current Office of Population Censuses and Surveys (OPCS) definitions (2, 3)
GP Practice Code (*gppractice*)	e.g. 'C87034'	A unique six character code ascribed to each GP practice in England (3, 4)

For peer review only

References

1. https://www.mrs.org.uk/pdf/postcodeformat.pdf.
2. Harmonised country specific ethnic group question(s) and dissemination of output(s) for use in social surveys and administrative data in England, Northern Ireland, Scotland and Wales https://gss.civilservice.gov.uk/policy-store/ethnicity/#great-britain.
3. Hospital Episode Statistics Data Dictionary https://digital.nhs.uk/data-and-information/data-tools-and-services/data-services/hospital-episode-statistics/hospital-episode-statistics-data-dictionary.
4. GP and GP practice related data https://digital.nhs.uk/services/organisation-data-service/data-downloads/gp-and-gp-practice-related-data.

For peer review only

BMJ Open

A national administrative record linkage between specialist community drug and alcohol treatment data (The National Drug Treatment Monitoring System (NDTMS)) and inpatient hospitalisation data (Hospital Episode Statistics (HES)) in England; Design, Method and Evaluation

Journal:	*BMJ Open*
Manuscript ID	bmjopen-2020-043540.R1
Article Type:	Original research
Date Submitted by the Author:	27-Oct-2020
Complete List of Authors:	Roberts , Emmert; Institute of Psychiatry Psychology and Neuroscience, National Addiction Centre; South London and Maudsley NHS Foundation Trust,<br>Doidge, James<br>Harron, Katie; London School of Hygiene and Tropical Medicine,<br>Hotopf, Matthew; King's College London (Institute of Psychiatry),<br>Knight, Jonathan; Public Health England<br>White, Martin; Public Health England<br>Eastwood, Brian; Public Health England<br>Drummond, Colin; Kings College London, Institute of Psychiatry
**Primary Subject Heading**:	Addiction
Secondary Subject Heading:	Epidemiology
Keywords:	PUBLIC HEALTH, EPIDEMIOLOGY, Substance misuse < PSYCHIATRY

SCHOLARONE™
Manuscripts

I, the Submitting Author has the right to grant and does grant on behalf of all authors of the Work (as defined in the below author licence), an exclusive licence and/or a non-exclusive licence for contributions from authors who are: i) UK Crown employees; ii) where BMJ has agreed a CC-BY licence shall apply, and/or iii) in accordance with the terms applicable for US Federal Government officers or employees acting as part of their official duties; on a worldwide, perpetual, irrevocable, royalty-free basis to BMJ Publishing Group Ltd ("BMJ") its licensees and where the relevant Journal is co-owned by BMJ to the co-owners of the Journal, to publish the Work in this journal and any other BMJ products and to exploit all rights, as set out in our licence.

The Submitting Author accepts and understands that any supply made under these terms is made by BMJ to the Submitting Author unless you are acting as an employee on behalf of your employer or a postgraduate student of an affiliated institution which is paying any applicable article publishing charge ("APC") for Open Access articles. Where the Submitting Author wishes to make the Work available on an Open Access basis (and intends to pay the relevant APC), the terms of reuse of such Open Access shall be governed by a Creative Commons licence – details of these licences and which Creative Commons licence will apply to this Work are set out in our licence referred to above.

Other than as permitted in any relevant BMJ Author's Self Archiving Policies, I confirm this Work has not been accepted for publication elsewhere, is not being considered for publication elsewhere and does not duplicate material already published. I confirm all authors consent to publication of this Work and authorise the granting of this licence.

For peer review only

Title: A national administrative record linkage between specialist community drug and alcohol treatment data (The National Drug Treatment Monitoring System (NDTMS)) and inpatient hospitalisation data (Hospital Episode Statistics (HES)) in England; Design, Method and Evaluation

Authors:

Dr Emmert Roberts, National Addiction Centre and the Department of Psychological Medicine, Institute of Psychiatry, Psychology and Neuroscience, Kings College London, South London and the Maudsley NHS Foundation Trust and Public Health England

Dr James C. Doidge, Intensive Care National Audit & Research Centre

Dr Katie L. Harron, Great Ormond Street Institute of Child Health, University College London, London, UK

Professor Matthew Hotopf, Department of Psychological Medicine, Institute of Psychiatry, Psychology and Neuroscience, Kings College London and the South London and the Maudsley NHS Foundation Trust

Jonathan Knight, Public Health England

Martin White, Public Health England

Dr Brian Eastwood*, Public Health England

Professor Colin Drummond*, National Addiction Centre, Institute of Psychiatry, Psychology and Neuroscience, Kings College London and the South London and the Maudsley NHS Foundation Trust

* Both authors contributed equally

Corresponding author:

Dr Emmert Roberts
National Addiction Centre, Institute of Psychiatry, Psychology and Neuroscience, King's College London

For peer review only - http://bmjopen.bmj.com/site/about/guidelines.xhtml

+447754156145
emmert.roberts@kcl.ac.uk

The corresponding author has the right to grant on behalf of all authors and does grant on behalf of all authors, an exclusive licence (or non-exclusive for government employees) on a worldwide basis to the BMJ Publishing Group Ltd to permit this article (if accepted) to be published in BMJ editions and any other BMJPGL products and sublicences such use and exploit all subsidiary rights, as set out in our licence

Word Count: 4121 (Max 4000)

For peer review only

Abstract (Word Count: 299; Max 300)

Objectives: The creation and evaluation of a national record linkage between substance misuse treatment, and inpatient hospitalisation data in England.

Design: A deterministic record linkage using personal identifiers to link the National Drug Treatment Monitoring System (NDTMS) curated by Public Health England (PHE), and Hospital Episode Statistics Admitted Patient Care (HES APC) curated by NHS Digital.

Setting and participants: Adults accessing substance misuse treatment in England between 1st April 2018 and 31st March 2019 (n=268,251) were linked to inpatient hospitalisation records available since 1st April 1997.

Outcome measures: Using a gold-standard subset, linked using NHS number, we report the overall linkage sensitivity and precision. Predictors for linkage error were identified, and inverse probability weighting was used to interrogate any potential impact on the analysis of length of hospital stay.

Results: 79.7% (n=213,814) people were linked to at least one HES record, with an estimated overall sensitivity of between 82.5% and 83.3%, and a precision of between 90.3% and 96.4%. Individuals were more likely to link if they were female, white, and aged between 46 and 60. Linked individuals were more likely to have an average length of hospital stay $\geq$ 5 days if they were male, older, had no fixed residential address, or had problematic opioid use. These associations did not change substantially after probability weighting, suggesting they were not affected by bias from linkage error.

Conclusions: Linkage between substance misuse treatment and hospitalisation records offers a powerful new tool to evaluate the impact of treatment on substance related harm in England. Whilst linkage error can produce misleading results, linkage bias appears to have little effect on the association between substance misuse treatment and length of hospital admission. As subsequent analyses are conducted, potential biases associated with the linkage process should be considered in the interpretation of any findings.

For peer review only - http://bmjopen.bmj.com/site/about/guidelines.xhtml

Strengths and limitations of this study

- This record linkage represents the first study of its kind to link centralised national level substance misuse treatment data and inpatient hospitalisation records
- No single unique identifier, such as NHS number, is routinely collected within NDTMS and fewer personal identifiers are routinely collected than in other UK government held datasets
- The limited availability of personal identifiers results in an increased risk of both false and missed matches, which could potentially affect the validity of any subsequently conducted analyses
- Linkage error did not appear to lead to systematic bias and misestimation of sociodemographic and clinical factor associations with the average length of hospital stay

For peer review only

Introduction

Routinely collected administrative data from the health and social care sector is increasingly used to both inform public health policy, and to generate research. Whilst several initiatives across the United Kingdom (UK) have used national record linkages to further population-level understanding in specific disease areas, (1) the ambition to harness record linkage as a means of improving health outcomes for people with drug and alcohol misuse has not been fully realised.

The number of people accessing specialist alcohol treatment has fallen by 19% between 2013 and 2017, while the number hospital admissions in which alcohol was recorded as a contributory factor has increased by 5% in the same timeframe. (2-4) Given this context, a recent report from the United Kingdom (UK) Department of Health and Social Care (DHSC) identified an urgent need to estimate the impact of specialist drug and alcohol treatment on acute care resource usage and substance-related harm. (5) The report posits that this goal may be achieved through detailed analysis of linked individual-level hospitalisation and substance misuse treatment data, which could '…*generate evidence to quantify the impact on health services utilisation before and after successful treatment*'. (5)

The National Drug Treatment Monitoring System (NDTMS) is the centralised database, collated and maintained by Public Health England (PHE), which receives monthly input from all local authority commissioned community drug and alcohol services in England. (6) This contains individual-level data on an individual's sociodemographic characteristics (date of birth, gender, ethnicity, housing status etc.), diagnostic characteristics, including the quantity and frequency of individuals' substance use, and treatment characteristics including frequency and type of contact with treatment services, the interventions received, and measures of treatment success. Hospital Episode Statistics (HES) is the centralised repository, collated and maintained by NHS Digital, which collects all information pertaining to National Health Service (NHS) hospitalisation in England and Wales. (7) The HES Admitted Patient Care (APC) database is one of the main administrative databases operating under the umbrella of HES and covers all NHS inpatient admissions, including any admission to private or third sector hospitals subsequently reimbursed by the NHS. (8) As such HES APC is estimated to contain > 99% of all inpatient hospital activity in England. (9) An inpatient hospital admission includes any secondary care-based activity requiring a hospital bed, thus includes day cases, and both planned and emergency admissions, in physical and mental health settings. HES APC does not cover accident and emergency

For peer review only - http://bmjopen.bmj.com/site/about/guidelines.xhtml

(A&E, emergency department) attendances, nor outpatient bookings, these data being held in separate HES databases.

Although NDTMS has been previously linked with mortality data from the Office of National Statistics (ONS), and the Police National Computer (PNC), (10-12) the lack of linkage between NDTMS and inpatient hospitalisation data limits the capacity to evaluate the impact of specialist drug and alcohol treatment on individual and regional rates of hospitalisation. International efforts have been made to facilitate record linkage of national databases in order to evaluate substance misuse outcomes, (13) however previous studies have often lacked access to national level data on substance misuse treatment, due in part to fragmented healthcare delivery systems, or lack of a centralised data repository. As centralised national databases exist for both hospitalisation and substance misuse treatment in England, we sought to link these two databases to inform drug and alcohol policy and research.

In this report we describe the process of record linkage and aim to evaluate the linkage quality and its potential impact on any subsequently conducted analyses. We believe this record linkage may result in the largest cross-sectional and longitudinal substance misuse database globally, and as such could become a resource which is able to support a large number of analytic outputs with the aim of improving the lives of those with substance use disorders.

For peer review only

Methods

Patients accessing publicly-funded specialist drug and alcohol treatment services in England provide written consent to share their information with NDTMS, and are informed that NDTMS records may be linked with data from specifically sanctioned UK government-held databases, including HES. (14) Over 98% of patients provide consent, (15) and the nature of this consent states that any record linkage would be undertaken by PHE, and that individuals may opt out at any time from having their records used within NDTMS. Approval to conduct the linkage was granted under regulation 3 of the Health Service (Control of Patient Information) Regulations 2002, (16) following review by the PHE Caldicott Advisory Panel (CAP) (Ref: CAP-2019-06).

Patient and Public Involvement

The study benefited throughout from discussion with the South London and the Maudsley (SLaM) Biomedical Research Centre (BRC) Data Linkage Service User and Carer Advisory Group, and the PHE Alcohol Treatment Expert Group which includes experts with lived experience. The former group represents a regular meeting of people whom have an interest in projects involving data linkage, and who have lived experience of mental health diagnoses, including substance use disorders. They receive on-going training on data matching processes, and hence can make recommendations on the acceptability of suggested data flows. The current proposal was presented in June 2018, and there was group-wide acknowledgement of the importance of the proposed linkage, based on personal experience of treatment experiences in drug and alcohol services. The group were content with the linkage methodology proposed, including the use of patient identifiers. Both groups will remain involved in subsequent analysis plans from any resultant linked data.

Linkage methods

Record linkage is the process of bringing together information pertaining to the same individual (or entity) from different databases. Linkage applies a set of criteria to determine whether or not records belong to the same individual, and aims to assess the true match status of each record pair: either a 'match' i.e. records belong to the same individual, or a 'non-match' i.e. records belong to different individuals. If record pairs are misclassified, error may be introduced as either 'false matches' i.e. records from different individuals link erroneously, or 'missed matches' i.e. records from the same individual fail to link. Introduction of bias from linkage error, particularly if risk factors for important outcomes are

For peer review only - http://bmjopen.bmj.com/site/about/guidelines.xhtml

associated with error rates, can impact the validity of findings derived from linked data. (17, 18) This is more likely to occur if datasets do not have a unique identifier in common. (19)

We selected all NDTMS records for adults accessing specialist drug or alcohol treatment in England between 1st April 2018 and 31st March 2019 as the test linkage population. The structure of the test linkage NDTMS data is such that one record represents one unique adult (n=268,251). (20) This was matched against all HES APC records available since database inception on 1st April 1997. The structure of the HES APC data is such that the same unique individual has multiple records, one for each hospital admission episode, with unique individuals identified by a specific variable, the HESID, which is assigned by NHS Digital (n=390,642,220 records; n=67,378,943 unique individuals). (21) As not all individuals presenting to drug and alcohol services will have been admitted to hospital, we did not expect all NDTMS records to match with HES APC. No unique person identifiers, such as NHS number, were shared between both databases but a number of personal demographic and geographic identifiers were available for matching. Identifiers were harmonised to maintain a consistent format across the two databases, which included harmonising string length, use of spaces, capitalisation, and hyphens. Five variables were available for matching; an individual's date of birth (DOB), sex, postcode, ethnicity and GP practice. Full variable descriptions can be found in the online supplementary material.

A Structured Query Language (SQL) algorithm was designed to facilitate NDTMS to HES APC linkage. Initial data cleaning in both datasets included the conversion of all missing or non-valid data to null values and the collapse of all postcodes relating to a no fixed abode status (NFA) into a single value. All NDTMS records contained a validly coded value for DOB and sex whilst 96.3% had a validly coded postcode, 94.7% a validly coded ethnicity and 18.4% a validly coded GP practice. NDTMS records that had missing or invalid postcodes (n=10,011, 3.7%) were excluded from linkage, as a combination of sex, postcode and date of birth was the minimum - but not necessarily sufficient - data required to uniquely identify an individual. Of the remaining n=258,240 NDTMS records n=6,878 (2.7%) shared the same combination of dob, sex and postcode, of which n=164 (2.4%) did not have a valid entry for either ethnicity or GP practice.

Matching was based on an exact match for each of the five variables described above, and conducted hierarchically in four stages as below:

Stage 1: Exact match on dob, sex, postcode, ethnicity and GP practice

For peer review only - http://bmjopen.bmj.com/site/about/guidelines.xhtml

Stage 2: Exact match on dob, sex, postcode and GP practice

Stage 3: Exact match on dob, sex, postcode and ethnicity

Stage 4: Exact match on dob, sex and postcode

When records matched, they were removed from the dataset and not included in subsequent matching stages. As both databases are longitudinal, it was possible that several different values for postcode, and GP practice were recorded for each individual over time. Where more than one unique value was available the hierarchical algorithm attempted to link NDTMS records to HES APC records sequentially starting with the most recent value for each variable. All resulting records that linked with multiple records from the other dataset were removed and treated as non-links.

Gold-standard subsample

A small subset of people in the full NDTMS sample (n=1,328), who to date were taking part in the PHE individual placement and support (IPS) trial, (22) had consented to make their unique ten-digit NHS number available. As NHS number is also coded within HES APC, this was used as a single unique identifier, common to both datasets, to facilitate linkage within this 'gold-standard' sample of individuals in NDTMS who had their NHS number available. The sociodemographic and clinical characteristics of the full NDTMS sample and the 'gold-standard' NDTMS sample are available in the online supplementary material as table S1.

Using the 'gold-standard' sample the linkage rate was calculated as the percentage of NDTMS individuals linked to any HES APC record first by exact matching on only NHS number, and secondly using the four-stage deterministic algorithm described above. The results were evaluated to determine the missed match rate, and the overall linkage precision, i.e. the proportion of links that are true.

Individuals linked using their NHS number were deemed to have been definitely hospitalised within their lifetime. Within this sample, individuals that were linked and not linked using the four-stage algorithm were compared to estimate rates of missed links. To allow for variation in patient characteristics and data quality between data providers, as well as between individuals, we used multilevel logistic regression with nesting of individuals within local authority commissioned treatment services and match status in NDTMS as the binary outcome (match=1, non-match=0). Model fit was examined using a likelihood ratio test comparing the multilevel model to a fixed-effects logistic model which did not account for nesting of individuals. We explored any association between match status and NDTMS

For peer review only - http://bmjopen.bmj.com/site/about/guidelines.xhtml
For peer review only

sociodemographic (e.g. sex, age, ethnicity, NFA status, and Index of Multiple Deprivation (IMD)), and clinical factors (e.g. the misused substance/s for which the person entered treatment). For modelling purposes ethnicity was recoded into the binary categories of white and non-white, (23) and probability estimates of matching as a function of the independent variables were generated.

Analysis of linkage error

Challenges exist to assess the impact of linkage error when the outcome in question may not have been experienced by all people in the sample to be matched. When linking an individual's NDTMS records to HES APC it is difficult to know which matches have been missed as the HES database by design will only capture information about individuals who have been hospitalised. As such non-links could be due to an individual never having been admitted to hospital or being a missed match. (24, 25)

For each unique linked individual, a binary outcome of their average length of hospital stay ($\geq$ 5 days=1, < 5 days=0) was created to assess bias due to linkage error. This was chosen as it is clinically relevant, reflecting the current UK average length of hospital stay per person, and is recorded for all people within HES APC. (7) Using the estimated probability of matching from the 'gold-standard' analysis, we created a weight that was inversely proportional to the probability of being linked to HES APC data using the four-stage algorithm. These weights were subsequently assigned to each linked individual, as per standard methods to account for non-response bias in cross-sectional and cohort studies. (26, 27) Univariable multilevel logistic regression was used within the 'gold-standard' sample to examine the association between independent variables and the average length of hospital stay. Estimates were generated using the 'unbiased' linked sample matched using NHS number, and these were then compared to estimates obtained using the 'biased' linked sample matched using the four-stage algorithm. The model applied to the 'biased' sample was firstly conducted without any weighting, secondly conducted incorporating the inverse probability weights to examine if this corrected any linkage error, and thirdly conducted weighted according to the odds of having sufficient matching data.

Data Access

Whilst access to the linked dataset is only available within PHE, subject to approval, extracts of NDTMS are available to researchers through the Office of Data Release (ODR) at PHE,

For peer review only - http://bmjopen.bmj.com/site/about/guidelines.xhtml

(28) and extracts of HES APC are available through the Data Access Request Service (DARS) at NHS Digital. (29)

The linkage was conducted using SQL Server Management Studio version 18.4. Additional analyses were conducted using STATA MP version 15.1, with the significance level set at 0.05.

For peer review only

Results

The overall matching for a unique person within the full NDTMS sample (n=268,251) to a HES APC hospitalisation record generated n=213,814 linked records, representing a linkage rate of 79.7%. The proportion linked according to the matching stages described above were: stage one: 10.7%, stage two: 5.7%, stage three: 72.5%, and stage four: 11.1%.

Gold-standard subsample

The overall matching for a unique person within NDTMS to a HES APC hospitalisation record using the 'gold-standard' subset of people with an NHS number available in NDTMS, generated n=1,153 linked records using NHS number, representing a linkage rate of 86.6%. Using the four-stage algorithm within the 'gold-standard' population generated n=1,053 linked records with a linkage rate of 79.3%. Although this was lower than the NHS number match rate this suggests that the majority of unlinked records were true non-links (i.e. individuals who had not previously been hospitalised) and not missed matches. Of the n=1,053 records linked using the four-stage algorithm, 102 were not linked by the gold standard. These included n=36 records that disagreed on NHS number and were therefore assumed to be false links, and 66 that had missing or invalid NHS numbers and could represent either false links or links missed by the gold standard. These two possibilities suggest a precision of between 90.3% and 96.4%, respectively. Of the n=1,153 records matched using the NHS number n=202 were not matched by the four-stage algorithm suggesting a sensitivity of between 82.5% and 83.3%, respectively.

Table one summarises the associations between sociodemographic and clinical variables and linkage by four-stage algorithm within the gold-standard subsample who linked to HES APC via their NHS number (n=1,153). Within this sample we compared individuals who were classified as linked or non-linked using the four-stage algorithm, an adjusted odds ratio (aOR) greater than 1 denoting increased odds of successful linkage when compared to the reference value. In the adjusted model, we found significant differences in the odds of linking for sex, age and ethnicity. There was strong evidence that when compared with women, men were significantly less likely to link to HES APC (aOR 0.48, 95%CI 0.30 to 0.79, p=0.003), when compared to those aged between 18-30, those aged between 46-60 were significantly more likely to link (aOR 2.28, 95%CI 1.08 to 4.82, p=0.03), and when compared to people of a white ethnicity, people with a non-white ethnicity were significantly less likely to link (aOR 0.35, 95%CI 0.20 to 0.63, p<0.001). The multilevel model was significantly

For peer review only - http://bmjopen.bmj.com/site/about/guidelines.xhtml

superior to the fixed-effects logistic model ($p<0.001$), with an intraclass correlation coefficient (ICC) of 0.13 (95%CI 0.04 to 0.36).

Analysis of linkage error

Weighting the probability of being linked to HES APC data using the four-stage algorithm demonstrated a correction of linkage bias within the 'gold-standard' sample, the results of which are summarised in table S2 in the online supplementary material.

The full linked sample had a total of 1,624,152 inpatient hospital admissions since HES database inception in April 1997 until January 2020, with a total time spent in hospital of 14,461 years, and an overall average length of hospital stay of three days. Table two summarises the associations between sociodemographic and clinical variables and the average length of hospital admission for linked individuals differentiated into those with an average length of hospital admission < 5 days and those with an average length of hospital admission ≥ 5 days. An adjusted odds ratio (aOR) greater than 1 denotes increased odds of an average length of hospital admission ≥ 5 days when compared to the reference value. In the adjusted model, we found significant differences in the average length of hospital admission across the majority of studied sociodemographic and clinical factors. There were no substantial differences between the estimates generated from the adjusted models following inverse probability or sufficient matching data weighting. The multilevel model was significantly superior to the fixed-effects logistic model ($p<0.001$), with an ICC of 0.02 (95%CI 0.01 to 0.02).

For peer review only - http://bmjopen.bmj.com/site/about/guidelines.xhtml

Discussion

Using deterministic matching, a national longitudinal and cross-sectional dataset was built between NDTMS specialist community drug and alcohol treatment data and HES hospitalisation data in England, providing a linkage for 213,814 adults (79.7% of the full NDTMS cohort) to their inpatient hospital records. Using our linkage algorithm there were significant differences in the sociodemographic and clinical characteristics between the linked and non-linked samples, with individuals more likely to link if they were female, white, and aged between 46 and 60 years old. Using the linked data, we were able to demonstrate that individuals were more likely to have an increased average length of hospital stay if they were male, older, had no fixed residential address, and had problematic opioid use. These effects did not change substantially following inverse probability weighting, suggesting they were not driven by bias from linkage error.

Analysis of Linkage Biases

Very few studies have examined linkage error in the context of people with substance use disorders. Using our deterministic algorithm, n=54,437 (20.3%) of individuals were not linked to HES APC hospitalisation records. Linkage of the gold-standard sample suggests that approximately two thirds of these are true non-links (i.e. arising because the individual had never been hospitalised and therefore had no HES record), and the remaining third are missed matches. When using the 'gold-standard' sample 86.6% of records matched using NHS number, as such 86.6% is likely to estimate the overall true match rate. We can thus infer that a roughly similar percentage of the n=10,011 NDTMS records with insufficient matching data should match and are therefore genuine missed-matches in the total sample (n=8,670). Based on our linkage sensitivity these 8,670 records constitute just under half of the total number of likely missed match records when using the four-stage algorithm. The sociodemographic and clinical characteristics of this cohort can be found in the online supplementary material as table S3, and when compared to the NDTMS cohort with sufficient matching data, demonstrate a substantially lower odds of having sufficient matching data if individuals were male, younger, and problematic opioid users. This indicates a higher likelihood of missed matches within these groups which is in accordance with the reduced odds of linkage for male, younger and non-white individuals observed using the four-stage algorithm in the 'gold-standard' sample.

We found that older age groups were more likely to link which may reflect a greater availability of accurate personal identifiers in the records of this population as by living longer

For peer review only - http://bmjopen.bmj.com/site/about/guidelines.xhtml

they have had greater potential exposure to drug and alcohol services compared to other age groups, and an increased number of hospitalisation records, and therefore potentially more values of matching variables. Previous research has suggested that individuals from black and ethnic minorities are more likely to have administrative records with inaccurately recorded dates of birth and higher levels of residential instability, which may be applicable to this sample, and partially account for the reduced likelihood in of linkage compared to white individuals. (30) It is reassuring however that in our sample, linkage biases do not appear to have significant effect on the associations between substance misuse and average length of hospital stay.

Strengths and limitations of the matching methods and evaluation

This represents the first study of its kind to link centralised national level substance misuse treatment data and inpatient hospitalisation records, and provides an example of how potential non-random loss between routinely collected administrative datasets can be adjusted for by weighting techniques. (31) As we had access to complete source data records, we were able to demonstrate that linkage error did not appear to lead to systematic bias and misestimation of sociodemographic and clinical factor associations with average length of hospital stay. It should be noted that in order to evaluate potential linkage bias within this paper we only report a single healthcare outcome. Following evaluation for potential linkage bias interrogation of the resultant dataset will be possible to address a number of key research and policy questions. There are also a number of limitations. Due to the previous practice of the UK Home Office compiling full names and addresses of all registered addicts in its 'Index of Addicts', (32) and more generally the stigma experienced by people with substance use disorders, NDTMS is careful to collect only the minimum amount of personal identifier information it deems necessary to balance the need for population surveillance, with legitimate concerns about individual identification. An unfortunate consequence, however, is that no single unique identifier, such as NHS number, is routinely collected within NDTMS and the personal identifiers which are collected are typically fewer than in other UK government held datasets. This creates a unique problem for NDTMS data linkage, which is compounded by the fact that, when compared to the general population, individuals within NDTMS are also less likely to be registered with a GP, more likely to not have a residential address, and potentially have an interest in providing non-accurate personal identification information to drug and alcohol services. All of the above reasons may contribute to the observed increased rate of false and missed matches compared to other national data linkages. (31) Nevertheless, a national centralised data repository for substance misuse treatment presents a unique opportunity to link with other

For peer review only - http://bmjopen.bmj.com/site/about/guidelines.xhtml

health and social care record systems, provided consent is given by those individuals, in an attempt to improve the lives of people with substance use disorders. This paucity of available personal identifiers results in an increased risk of both false and missed matches, particularly at lower confidence matching stages and these limitations could have led to our match rate being an overestimation of the linkage performance. As such in order to minimise the risk of false matches, records that linked with multiple different unique records from either dataset were removed and treated as non-links. These could reflect imperfect internal linkage or deduplication of NDTMS or HES; i.e. these could be true multiple links and this linkage strategy could increase the rate of missed matches. When 'gold-standard' data are used to assess linkage quality this is assumed to be representative in terms of the distribution of the quality of matching and analysis variables. Although our 'gold-standard' dataset unique person identifier is NHS number we cannot exclude the possibility there may be coding errors within NHS numbers and the dataset may not represent the remainder of records. Although there was a significantly lower linkage rate using our algorithm compared to using NHS number within the gold-standard sample (79.7% vs 86.6%), differences in ethnicity and age between the full and gold-standard NDTMS samples partially explain this difference, but do not appear to contribute significant bias due to linkage error.

Implications

This linkage between substance misuse treatment and hospitalisation records offers a new powerful tool to evaluate the impact of specialist treatment on alcohol and drug related harm in England. Through its interrogation, and via additional sanctioned linkage to datasets from other government departments, e.g. the Department for Work and Pensions (DWP), this data may hopefully be able to provide insight and knowledge to improve the lives of people with substance use disorders. Whilst biases due to linkage error may produce misleading results in our sample, linkage biases appear to have little effect on the association between drug and alcohol treatment and length of hospital admission. However, without ongoing ability to probe information within the source data, potential linkage error could be introduced without future analysts being aware that there was need for it to be accounted for.

In time, we hope this resource will generate a wide network of granular data and analytical expertise, which can be used to inform both commissioning and service provision to better meet the needs of people with substance use disorders in England. The immediate next steps are to evaluate the most common reasons for hospital admission within the cohort of people accessing drug and alcohol treatment and to assess the impact of engagement in, and successful completion of, drug and alcohol treatment on individual and national rates

For peer review only - http://bmjopen.bmj.com/site/about/guidelines.xhtml

hospitalisation. It is important to note that as subsequent analyses of the resultant linked dataset are conducted, any potential bias associated with the linkage process should always be considered in the interpretation of any findings.

For peer review only

Contributorship statement:

All authors meet the ICMJE criteria for authorship:

Dr Roberts formulated the research question, designed and carried out the study, analysed the data and drafted the article
Dr Doidge contributed to the study design, data analysis and writing the article
Dr Harron contributed to the study design, data analysis and writing the article
Professor Hotopf contributed to the formulation of the research question, study design, data analysis and writing the article
Mr Knight contributed to the study design, data interpretation and writing the article
Mr White contributed to the study design, data interpretation and writing the article
Dr Eastwood contributed to the formulation of the research question, study design, data analysis and writing the article
Professor Drummond contributed to the formulation of the research question, study design, data analysis and writing the article

Competing Interest Statement:

All authors have completed the ICJME Unified Competing Interest form (available on request from the corresponding author) and declare: no support from any organisation for the submitted work; no financial relationships with organisations that might have an interest in the submitted work in the previous three years, no other relationships or activities that could appear to have influenced the submitted work.

Professor Hotopf is principal investigator of RADAR-CNS consortium - a public private partnership in collaboration with five pharma companies - Janssen, Biogen, UCB, MSD and Lundbeck, outside of the submitted work.

Role of the funding source:

This paper represents independent research funded by the Medical Research Council (MRC), as part of the corresponding author's MRC Addiction Research Clinical (MARC) Fellowship. The research was part funded by the NIHR Biomedical Research Centre at South London and Maudsley NHS Foundation Trust and King's College London, and by the NIHR Collaboration for Leadership in Applied Health Research and Care South London (NIHR CLAHRC South London) now recommissioned as NIHR Applied Research

For peer review only - http://bmjopen.bmj.com/site/about/guidelines.xhtml

Collaboration South London, and both CD and MH receive funding from an NIHR Senior Investigator award. KH is funded in part by the Wellcome Trust (grant number 212953/Z/18/Z) and benefits from infrastructure funded by NIHR GOSH BRC and Health Data Research UK. The funders had no contribution to the study design; in the collection, analysis, and interpretation of data; in the writing of the report; and in the decision to submit the article for publication. All authors were independent from funders had full access to all of the data (including statistical reports and tables) in the study and take responsibility for the integrity of the data and the accuracy of the data analysis. The views expressed are those of the authors and not necessarily those of the MRC, the National Health Service (NHS), the NIHR, Public Health England (PHE) or the Department of Health and Social Care (DHSC).

Data sharing statement:

Whilst access to the linked dataset is only available within Public Health England, subject to approval, extracts of NDTMS are available to researchers through the Office of Data Release (ODR) at PHE, and extracts of HES APC are available through the Data Access Request Service (DARS) at NHS Digital.

Transparency declaration:

The corresponding author (the manuscript's guarantor) affirms that the manuscript is an honest, accurate, and transparent account of the study being reported; that no important aspects of the study have been omitted; and that any discrepancies from the study as originally planned have been explained.

For peer review only

References

1. Enabling Data Linkage to Maximise the Value of Public Health Research Data: full report https://wellcome.ac.uk/sites/default/files/enabling-data-linkage-to-maximise-value-of-public-health-research-data-phrdf-mar15.pdf.
2. Statistics on Alcohol England 2018 https://files.digital.nhs.uk/60/B4D319/alc-eng-2018-rep.pdf.
3. Adult substance misuse statistics rom the National Drug Treatment Monitoring System (NDTMS) 1 April 2017 to 31 March 2018 https://assets.publishing.service.gov.uk/government/uploads/system/uploads/attachment_data/file/752993/AdultSubstanceMisuseStatisticsfromNDTMS2017-18.pdf.
4. PHE inquiry into the fall in numbers of people in alcohol treatment: findings Published https://www.gov.uk/government/publications/alcohol-treatment-inquiry-summary-of-findings/phe-inquiry-into-the-fall-in-numbers-of-people-in-alcohol-treatment-findings#the-inquiry-process. 2018.
5. Hill-McManus D, Stone T, Ally A, Pryce RE, Gillespie D, Buykx P, et al. An Evidence-Based Model for Estimating Requirements for Specialist Alcohol Treatment Capacity in England The Specialist Treatment for Alcohol Model (STreAM) Version 1.0. 2016.
6. National Drug Treatment Monitoring System https://digital.nhs.uk/data-and-information/information-standards/information-standards-and-data-collections-including-extractions/publications-and-notifications/standards-and-collections/dcb0107-national-drug-treatment-monitoring-system.
7. Hospital Admitted Patient Care Activity https://digital.nhs.uk/data-and-information/publications/statistical/hospital-admitted-patient-care-activity.
8. Herbert A, Wijlaars L, Zylbersztejn A, Cromwell D, Hardelid P. Data resource profile: hospital episode statistics admitted patient care (HES APC). International journal of epidemiology. 2017;46(4):1093-i.
9. Healthcare across the UK: A comparison of the NHS in England, Scotland, Wales and Northern Ireland https://www.nao.org.uk/wp-content/uploads/2012/06/1213192.pdf.
10. Willey H, Eastwood B, Gee IL, Marsden J. Is treatment for alcohol use disorder associated with reductions in criminal offending? A national data linkage cohort study in England. Drug and alcohol dependence. 2016;161:67-76.
11. White M, Burton R, Darke S, Eastwood B, Knight J, Millar T, et al. Fatal opioid poisoning: a counterfactual model to estimate the preventive effect of treatment for opioid use disorder in England. Addiction. 2015;110(8):1321-9.
12. Drug and alcohol addiction, and obesity: effects on employment outcomes https://www.gov.uk/government/publications/drug-and-alcohol-addiction-and-obesity-effects-on-employment-outcomes.
13. Peacock A, Chiu V, Leung J, Dobbins T, Larney S, Gisev N, et al. Protocol for the Data-Linkage Alcohol Cohort Study (DACS): investigating mortality, morbidity and offending among people with an alcohol-related problem using linked administrative data. BMJ Open. 2019;9(8):e030605.
14. NDTMS: consent and confidentiality guidelines https://www.gov.uk/government/publications/confidentiality-guidance-for-drug-and-alcohol-treatment-providers-and-clients.

For peer review only - http://bmjopen.bmj.com/site/about/guidelines.xhtml

15. Marsden J, Eastwood B, Bradbury C, Dale-Perera A, Farrell M, Hammond P, et al. Effectiveness of community treatments for heroin and crack cocaine addiction in England: a prospective, in-treatment cohort study. The Lancet. 2009;374(9697):1262-70.
16. The Health Service (Control of Patient Information) Regulations 2002 http://www.legislation.gov.uk/uksi/2002/1438/contents/made.
17. Lariscy JT. Differential record linkage by Hispanic ethnicity and age in linked mortality studies: implications for the epidemiologic paradox. Journal of aging and health. 2011;23(8):1263-84.
18. Doidge JC, Harron KL. Reflections on modern methods: linkage error bias. International Journal of Epidemiology. 2019.
19. Harron K, Goldstein H, Dibben C. Methodological developments in data linkage: John Wiley & Sons; 2015.
20. Substance misuse treatment for adults: statistics 2018 to 2019 https://www.gov.uk/government/statistics/substance-misuse-treatment-for-adults-statistics-2018-to-2019.
21. Replacement of the HES Patient ID (HESID) https://www.cl.cam.ac.uk/~rja14/Papers/HESID_Replacement_Nov09.pdf.
22. Marsden J, Anders P, Clark H, Colocassis K, Eastwood B, Knight J, et al. Protocol for a multi-centre, definitive randomised controlled trial of the effectiveness of Individual Placement and Support for employment support among people with alcohol and drug dependence. Trials. 2020;21(1):1-12.
23. Marsden J, Eastwood B, Jones H, Bradbury C, Hickman M, Knight J, et al. Risk adjustment of heroin treatment outcomes for comparative performance assessment in E ngland. Addiction. 2012;107(12):2161-72.
24. Bohensky MA, Jolley D, Sundararajan V, Evans S, Pilcher DV, Scott I, et al. Data linkage: a powerful research tool with potential problems. BMC health services research. 2010;10(1):346.
25. Harron KL, Doidge JC, Knight HE, Gilbert RE, Goldstein H, Cromwell DA, et al. A guide to evaluating linkage quality for the analysis of linked data. International journal of epidemiology. 2017;46(5):1699-710.
26. Höfler M, Pfister H, Lieb R, Wittchen H-U. The use of weights to account for non-response and drop-out. Social psychiatry and psychiatric epidemiology. 2005;40(4):291-9.
27. Little RJ, Vartivarian S. On weighting the rates in non-response weights. Statistics in medicine. 2003;22(9):1589-99.
28. Accessing PHE data through the Office for Data Release https://www.gov.uk/government/publications/accessing-public-health-england-data/about-the-phe-odr-and-accessing-data.
29. Data Access Request Service (DARS) https://digital.nhs.uk/services/data-access-request-service-dars.
30. Hagger-Johnson G, Harron K, Goldstein H, Aldridge R, Gilbert R. Probabilistic linking to enhance deterministic algorithms and reduce linkage errors in hospital administrative data. Journal of innovation in health informatics. 2017;24(2):891.
31. Downs JM, Ford T, Stewart R, Epstein S, Shetty H, Little R, et al. An approach to linking education, social care and electronic health records for children and young people in South London: a linkage study of child and adolescent mental health service data. BMJ open. 2019;9(1):e024355.

For peer review only

32. Ghodse AH, Sheehan M, Taylor C, Edwards G. Deaths of drug addicts in the United Kingdom 1967-81. Br Med J (Clin Res Ed). 1985;290(6466):425-8.

For peer review only

Tables

Table one: Sociodemographic and clinical characteristics of the n=1,153 individuals in NDTMS linked to HES APC using NHS number characterised as either linked or non-linked to HES APC using the four-stage algorithm

		Linked Pairs n (%)	Non-Linked Residuals n (%)	OR (95% CI) for positive linkage	p value	aOR[1] (95%CI) for positive linkage	p value
**All**	**All**	951 (82.5)	202 (17.5)	-	-	-	-
**Sociodemographic**	**Sex**						
	Female	320 (86.5)	50 (13.5)	Reference		Reference	
	Male	631 (80.6)	152 (19.4)	0.66 (0.46-0.95)	0.03*	0.48 (0.30-0.79)	0.003*
	**Age in years (at presentation to D&A services)**						
	18-30	97 (81.5)	22 (18.5)	Reference		Reference	
	31-45	497 (79.1)	131 (20.9)	0.79 (0.46-1.36)	0.40	1.10 (0.56-2.19)	0.78
	46-60	336 (87.7)	47 (12.3)	1.53 (0.85-2.76)	0.16	2.28 (1.08-4.82)	0.03*
	60+	21 (91.3)	2 (8.7)	1.62 (0.34-7.83)	0.55	1.79 (0.33-9.62)	0.50
	**Deprivation (IMD) Quintile**						
	First (Most deprived)	328 (81.6)	74 (18.4)	Reference		Reference	
	Second	275 (85.1)	48 (14.9)	0.96 (0.62-1.49)	0.87	0.81 (0.50-1.32)	0.40
	Third	210 (90.5)	22 (9.5)	1.38 (0.77-2.47)	0.28	1.04 (0.55-1.98)	0.90
	Fourth	89 (89.9)	10 (10.1)	1.07 (0.49-2.34)	0.87	1.22 (0.46-3.23)	0.69
	Fifth (Least deprived)	17 (85.0)	3 (15.0)	1.14 (0.30-4.30)	0.85	0.49 (0.12-1.99)	0.32
	**Residential Status[2]**						
	Non NFA postcode	913 (81.9)	202 (18.1)	Reference		Reference	
	NFA postcode	38 (100.0)	0 (0.0)	-	-	-	-
	**Ethnicity[3]**						
	White	840 (88.9)	105 (11.1)	Reference		Reference	
	Non-white	80 (73.4)	29 (26.6)	0.42 (0.24-0.71)	<0.001*	0.35 (0.20-0.63)	<0.001*
**Clinical**	**Substance Misuse[4]**						
	Opioid	503 (77.3)	148 (22.7)	Reference		Reference	
	Alcohol only	311 (90.7)	32 (9.3)	2.41 (1.57-3.68)	<0.001*	1.57 (0.95-2.61)	0.08
	Non-opioid and alcohol	75 (91.5)	7 (8.5)	2.16 (0.95-0.4.93)	0.07	2.03 (0.76-5.44)	0.16
	Non-opioid only	62 (80.5)	15 (19.5)	1.12 (0.59-2.12)	0.73	0.98 (0.44-2.18)	0.96

* p<0.05; OR Odds Ratio; aOR Adjusted Odds Ratio; CI Confidence Interval; IMD Index of Multiple Deprivation

1 Adjusted for all other covariates listed in table; 2 Residential status was omitted from the model as all people with an NFA postcode were linked 3 Office of Population Censuses and Surveys (OPCS) categories A, B and C collapsed as white, all other OPCS categories (D-S) collapsed as non-white; 4 NDTMS categorisation **opioid**: clients with any mention of opioid use in any treatment episode during the year irrespective of other substances cited; **alcohol only:** clients who present with problems related to alcohol but no other substances; **non-opioid and alcohol:** clients with non-opioid drug *and* alcohol use problems (but *not* opioids) recorded in any treatment episode during the year; **non-opioid only:** clients who present for treatment related to non-opioid drug use but *not* opioids or alcohol

For peer review only

For peer review only - http://bmjopen.bmj.com/site/about/guidelines.xhtml

Table two: The odds of an average ≥ 5 day length of hospital admission in the 213,814 people in treatment at drug and alcohol services

		≥ 5 days average hospital admission length n (%)	< 5 days average hospital admission length n (%)	OR (95%CI)	aOR[1] (95%CI)	Inverse probability weighted (IPW) for missed links aOR[2] (95%CI)	Weighted for sufficient matching data aOR (95% CI)
**All**	**All**	25,814 (12.1)	188,000 (87.9)				
**Sociodemographic**	**Sex**						
	Female	6,715 (9.4)	65,114 (90.6)	Reference	Reference	Reference	Reference
	Male	19,099 (13.5)	122,886 (86.5)	1.51 (1.47-1.56)	1.42 (1.37-1.46)	1.42 (1.37-1.47)	1.42 (1.37-1.47)
	**Age in years (at presentation to D&A services)**						
	18-30	1,979 (7.1)	26,040 (92.9)	Reference	Reference	Reference	Reference
	31-45	10,804 (10.9)	88,392 (89.1)	1.58 (1.50-1.66)	1.46 (1.38-1.54)	1.46 (1.37-1.54)	1.46 (1.37-1.54)
	46-60	10,771 (14.7)	62,634 (85.3)	2.17 (2.06-2.28)	2.08 (1.96-2.20)	2.07 (1.95-2.20)	2.08 (1.96-2.20)
	60+	2,260 (17.1)	10,934 (82.9)	2.62 (2.46-2.80)	2.84 (2.65-3.06)	2.81 (2.61-3.04)	2.84 (2.63-3.06)
	**Deprivation (IMD) Quintile**						
	First (Most deprived)	8,340 (12.4)	58,831 (87.6)	Reference	Reference	Reference	Reference
	Second	6,988 (12.4)	49,543 (87.6)	0.99 (0.95-1.03)	1.01 (0.97-1.05)	1.01 (0.97-1.06)	1.01 (0.97-1.06)
	Third	4,560 (11.5)	35,324 (88.5)	0.94 (0.90-0.98)	0.98 (0.94-1.02)	0.98 (0.93-1.04)	0.98 (0.93-1.04)
	Fourth	3,045 (10.8)	25,250 (89.2)	0.90 (0.86-0.95)	0.95 (0.90-1.00)	0.95 (0.90-1.01)	0.95 (0.90-1.01)
	Fifth (Least deprived)	1,289 (10.0)	11,599 (90.0)	0.85 (0.79-0.91)	0.90 (0.84-0.97)	0.90 (0.84-0.97)	0.90 (0.85-0.97)
	**Residential Status**						
	Non NFA postcode	24,108 (11.8)	180,389 (88.2)	Reference	Reference	Reference	Reference
	NFA postcode	1,706 (18.3)	7,611 (81.7)	1.65 (1.57-1.75)	1.44 (1.27-1.63)	1.43 (1.24-1.65)	1.43 (1.24-1.65)
	**Ethnicity[3]**						
	White	22,808 (11.8)	170,772 (88.2)	Reference	Reference	Reference	Reference
	Non-white	2,462 (15.3)	13,650 (84.7)	1.20 (1.14-1.26)	1.19 (1.13-1.25)	1.18 (1.11-1.26)	1.19 (1.12-1.27)
	**Substance Misuse[4]**						
**Clinical**	Opioid	15,309 (14.3)	92,102 (85.7)	Reference	Reference	Reference	Reference
	Alcohol only	6,776 (10.4)	58,084 (89.6)	0.71 (0.69-0.74)	0.68 (0.66-0.70)	0.68 (0.65-0.72)	0.68 (0.64-0.71)
	Non-opioid and alcohol	2,055 (9.0)	20,820 (91.0)	0.58 (0.55-0.61)	0.67 (0.63-0.70)	0.67 (0.63-0.72)	0.67 (0.62-0.71)
	Non-opioid only	1,674 (9.0)	16,994 (91.0)	0.59 (0.56-0.62)	0.74 (0.70-0.79)	0.76 (0.70-0.82)	0.74 (0.69-0.80)

OR Odds Ratio; aOR Adjusted Odds Ratio; CI Confidence Interval; IMD Index of Multiple Deprivation

1 Adjusted for all other covariates listed in table; 2 Adjusted model with inverse probability weighting for matching included; 3 Office of Population Censuses and Surveys (OPCS) categories A, B and C collapsed as white, all other OPCS categories (D-S) collapsed as non-white; 4 NDTMS categorisation **opioid**: clients with any mention of opioid use in any treatment episode during the year irrespective of other substances cited; **alcohol only:** clients who present with problems related to alcohol but no other substances; **non-opioid and alcohol:** clients with non-opioid drug *and* alcohol use problems (but *not* opioids) recorded in any treatment episode during the year; **non-opioid only:** clients who present for treatment related to non-opioid drug use but *not* opioids or alcohol

For peer review only

Online Supplementary Material

Contents

Tables

S1: Differences between full NDTMS cohort and the ‘gold-standard’ NDTMS cohort with NHS number available

S2: The odds of an average ≥ 5 day length of hospital admission within the ‘gold-standard’ NDTMS cohort with NHS number available, in the ‘unbiased’ sample matched using NHS number and the ‘biased’ sample matched using the four-stage algorithm, following application of inverse probability weighting to correct for potential linkage bias

S3: Differences between NDTMS cohort that contains sufficient matching data (i.e. a validly coded dob, sex and postcode) and the NDTMS cohort that does not contain sufficient matching data

S4: Description of variables available for linkage

References

For peer review only

Tables

Table S1: Differences between full NDTMS cohort and the 'gold-standard' NDTMS cohort with NHS number available

		Full NDTMS Cohort n (%)	Cohort with NHS number n (%)	p value
**All**		268,251 (100.0)	1,328 (100.0)	
**Sociodemographic**	**Sex**			
	Female	83,015 (31.0)	393 (29.6)	0.29
	Male	185,236 (69.0)	935 (70.4)	
	**Age in years (at presentation to D&A services)**			
	18-30	36,068 (13.4)	143 (10.8)	0.04*
	31-45	127,635 (47.6)	720 (54.2)	
	46-60	89,643 (33.4)	438 (33.0)	
	60+	14,905 (5.6)	27 (2.0)	
	**Deprivation (IMD) Quintile**			
	First (Most deprived)	81,478 (33.2)	472 (39.9)	<0.001*
	Second	68,808 (28.0)	366 (29.4)	
	Third	47,139 (19.2)	271 (21.8)	
	Fourth	33,354 (13.6)	111 (8.9)	
	Fifth (Least deprived)	15,002 (6.1)	25 (2.0)	
	**Residential Status**			
	Non NFA postcode	255,515 (95.3)	1,286 (96.8)	0.01*
	NFA postcode	12,736 (4.7)	42 (3.2)	
	**Ethnicity**[1]			
	White	230,012 (90.6)	1,064 (87.5)	<0.001*
	Non-white	23,928 (9.4)	152 (12.5)	
**Clinical**	**Substance Misuse**[2]			
	Opioid	139,845 (52.1)	775 (58.4)	<0.001*
	Alcohol only	75,555 (28.2)	362 (27.3)	
	Non-opioid and alcohol	28,598 (10.7)	95 (7.1)	
	Non-opioid only	24,253 (9.0)	96 (7.2)	

* p<0.05; 1 Office of Population Censuses and Surveys (OPCS) categories A, B and C collapsed as white, all other OPCS categories (D-S) collapsed as non-white
2 NDTMS categorisation **opioid**: clients with any mention of opioid use in any treatment episode during the year irrespective of other substances cited; **alcohol only:** clients who present with problems related to alcohol but no other substances; **non-opioid and alcohol:** clients with non-opioid drug *and* alcohol use problems (but *not* opioids) recorded in any treatment episode during the year; **non-opioid only:** clients who present for treatment related to non-opioid drug use but *not* opioids or alcohol

For peer review only

Table S2: The conditional odds of an average ≥ 5 day length of hospital admission within the ‘gold-standard’ NDTMS cohort with NHS number available, in the ‘unbiased’ sample matched using NHS number and the ‘biased’ sample matched using the four-stage algorithm, following application of inverse probability weighting to correct for potential linkage bias

		‘Unbiased’ sample matched using NHS number		‘Biased’ sample matched using four-stage algorithm		
		n (%)	OR (95%CI)	n (%)	OR (95%CI)	Weighted OR[1] (95%CI)
	**All**	1,153 (100.0)	-	1,053 (100.0)	-	-
**Sociodemographic**	**Sex**					
	Female	370 (32.1)	Reference	336 (31.9)	Reference	Reference
	Male	783 (67.9)	1.27 (0.80-2.02)	717 (68.1)	1.33 (0.85-2.09)	1.26 (0.80-2.00)
	**Age in years (at presentation to D&A services)**					
	≤ 45	747 (64.8)	Reference	658 (62.5)	Reference	Reference
	> 45	406 (35.2)	1.07 (0.69-1.65)	395 (37.5)	1.17 (0.78-1.77)	1.11 (0.71-1.73)
	**Deprivation (IMD) Quintile**					
	First (Most deprived)	402 (37.4)	Reference	363 (35.7)	Reference	Reference
	All other quintiles	674 (62.6)	0.64 (0.42-1.00)	655 (64.3)	0.71 (0.47-1.08)	0.70 (0.40-1.10)
	**Residential Status**					
	Non NFA postcode	1,115 (96.7)	Reference	1,011 (96.0)	Reference	Reference
	NFA postcode	38 (3.3)	0.59 (0.13-2.51)	42 (4.0)	0.56 (0.13-2.35)	0.60 (0.12-2.49)
	**Ethnicity**[2]					
	White	945 (89.7)	Reference	915 (90.0)	Reference	Reference
	Non-white	109 (10.3)	1.74 (0.90-3.36)	102 (10.0)	2.19 (1.25-3.84)	1.89 (0.99-3.39)
**Clinical**	**Substance Misuse**[3]					
	Opioid	651 (56.5)	Reference	569 (54.0)	Reference	Reference
	All other drug categories	502 (43.5)	0.67 (0.43-1.04)	484 (46.0)	0.65 (0.43-0.99)	0.65 (0.42-1.00)

OR Odds Ratio; CI Confidence Interval; IMD Index of Multiple Deprivation

1 Model with inverse probability weighting for matching included; 2 Office of Population Censuses and Surveys (OPCS) categories A, B and C collapsed as white, all other OPCS categories (D-S) collapsed as non-white; 3 NDTMS categorisation **opioid**: clients with any mention of opioid use in any treatment episode during the year irrespective of other substances cited; **alcohol only:** clients who present with problems related to alcohol but no other substances; **non-opioid and alcohol:** clients with non-opioid drug *and* alcohol use problems (but *not* opioids) recorded in any treatment episode during the year; **non-opioid only:** clients who present for treatment related to non-opioid drug use but *not* opioids or alcohol

For peer review only

Table S3: Differences between NDTMS cohort that contains sufficient matching data (i.e. a validly coded dob, sex and postcode) and the NDTMS cohort that does not contain sufficient matching data

		NDTMS cohort that does contain sufficient matching data n (%)	NDTMS cohort that does not contain sufficient matching data n (%)	OR (95%CI)	aOR (95%CI)[1]
**All**		258,240 (100.0)	10,011 (100.0)	-	
**Sociodemographic**	**Sex**				
	Female	80,111 (31.0)	2,904 (29.0)	Reference	Reference
	Male	178,129 (69.0)	7,107 (71.0)	1.10 (1.05-1.15)	1.27 (1.15-1.40)
	**Age in years (at presentation to D&A services)**				
	18-30	35,446 (13.7)	622 (6.2)	Reference	Reference
	31-45	122,999 (47.6)	4,636 (56.3)	2.15 (1.97-2.34)	0.94 (0.83-1.06)
	46-60	85,425 (33.1)	4,218 (42.1)	2.81 (2.58-3.06)	0.61 (0.53-0.71)
	60+	14,370 (5.6)	535 (5.3)	2.12 (1.89-2.39)	0.52 (0.40-0.67)
	**Deprivation (IMD) Quintile**[2]				
	First (Most deprived)	81,478 (33.2)	-	-	-
	Second	68,808 (28.0)	-	-	-
	Third	47,139 (19.2)	-	-	-
	Fourth	33,354 (13.6)	-	-	-
	Fifth (Least deprived)	15,002 (6.1)	-	-	-
	**Residential Status**[2]				-
	Non NFA postcode	245,504 (95.1)	-	-	-
	NFA postcode	12,736 (4.9)	-	-	-
	**Ethnicity**[3]				
	White	228,023 (90.6)	1,989 (89.2)	Reference	Reference
	Non-white	23,688 (9.4)	240 (10.8)	1.16 (1.02-1.33)	0.84 (0.73-0.97)
**Clinical**	**Substance Misuse**[4]				
	Opioid	131,316 (50.9)	8,529 (85.2)	Reference	Reference
	Alcohol only	74,840 (29.0)	715 (7.1)	0.15 (0.14-0.16)	0.86 (0.77-0.95)
	Non-opioid and alcohol	28,202 (10.9)	396 (4.0)	0.22 (0.20-0.24)	1.07 (0.93-1.22)
	Non-opioid only	23,882 (9.3)	371 (3.7)	0.24 (0.22-0.27)	1.05 (0.91-1.22)

1 Adjusted for all other covariates listed in table 2 All records that did not contain sufficient matching data lacked validly coded postcodes, as such no IMD quintile or residential status values were available for this cohort. 3 Office of Population Censuses and Surveys (OPCS) categories A, B and C collapsed as white, all other OPCS categories (D-S) collapsed as non-white 4 NDTMS categorisation **opioid**: clients with any mention of opioid use in any treatment episode during the year irrespective of other substances cited; **alcohol only:** clients who present with problems related to alcohol but no other substances; **non-opioid and alcohol:** clients with non-opioid drug *and* alcohol use problems (but *not* opioids) recorded in any treatment episode during the year; **non-opioid only:** clients who present for treatment related to non-opioid drug use but *not* opioids or alcohol

For peer review only

Table S4: Description of variables available for linkage

Variable	Format	Notes
Date of Birth (*dob*)	'yyyymmdd'	
Sex (*sex*)	1=male; 2=female	
Sector Level Postcode (*postcode*)	e.g. 'S752' or 'NE177'	This contains the entire 'outcode' and the first character of the 'incode', each unique sector level postcode representing roughly 3000 households in England (1)
Ethnicity (*ethnicity*)	e.g. 'A'	16 categories A - S harmonised to current Office of Population Censuses and Surveys (OPCS) definitions (2, 3)
GP Practice Code (*gppractice*)	e.g. 'C87034'	A unique six character code ascribed to each GP practice in England (3, 4)

For peer review only

References

1. https://www.mrs.org.uk/pdf/postcodeformat.pdf.
2. Harmonised country specific ethnic group question(s) and dissemination of output(s) for use in social surveys and administrative data in England, Northern Ireland, Scotland and Wales https://gss.civilservice.gov.uk/policy-store/ethnicity/#great-britain.
3. Hospital Episode Statistics Data Dictionary https://digital.nhs.uk/data-and-information/data-tools-and-services/data-services/hospital-episode-statistics/hospital-episode-statistics-data-dictionary.
4. GP and GP practice related data https://digital.nhs.uk/services/organisation-data-service/data-downloads/gp-and-gp-practice-related-data.

For peer review only
